# RNA therapeutics in rheumatoid arthritis, systemic lupus erythematosus, and systemic sclerosis: a compartment-aware translational framework

**DOI:** 10.3389/fimmu.2026.1950465

**Published:** 2026-10-05

**Authors:** Yusif Afandizada, Hikmet Memmedov, Mushfig Orujov, Shahla Eyvazova, Shirkhan Amikishiyev

**Affiliations:** 1 NOBELab (Nucleosides, Oligonucleotides, and Biological Evaluation Laboratory), Division of Basic Medical Sciences, Faculty of Medicine and Health Sciences, Qarabağ University, Khankendi, Azerbaijan; 2 Division of Basic Medical Sciences, Faculty of Medicine and Health Sciences, Qarabağ University, Khankendi, Azerbaijan; 3 Division of Internal Medicine, Faculty of Medicine and Health Sciences, Qarabağ University, Khankendi, Azerbaijan

**Keywords:** antisense oligonucleotides, drug delivery, lupus nephritis, rheumatoid arthritis, RNA therapeutics, small interfering RNA, systemic lupus erythematosus, systemic sclerosis

## Abstract

Rheumatoid arthritis (RA), systemic lupus erythematosus/lupus nephritis (SLE/LN), and systemic sclerosis (SSc) remain major causes of chronic inflammation, disability, organ damage, and treatment burden despite therapeutic advances. RNA therapeutics offer programmable transcript suppression, network-level microRNA modulation, transient restoration of protective proteins, and targeting-ligand functions. Existing reviews largely catalogue modalities or individual delivery systems; this review instead asks whether a candidate forms an interpretable translational program across target biology, disease compartment, intended cell, productive intracellular access, direct target engagement, pathway pharmacodynamics, repeat-dose safety, and manufacturing feasibility. RA is primarily a synovial immune–stromal delivery problem in which RNA programs must also demonstrate an advantage over effective biologic or targeted synthetic therapies. SLE/LN offers interferon, B-cell, co-stimulatory, microRNA, messenger RNA (mRNA), and complement opportunities, but therapeutic nucleic acids and carriers must not intensify nucleic-acid sensing, interferon activity, or complement-associated injury. SSc combines strong antifibrotic RNA biology with an accessible skin proof-of-mechanism window, whereas lung, vascular, and internal-organ programs remain more delivery-limited. We separate human pathway validation, RNA-platform evidence outside the target disease, disease-specific RNA evidence, and patient efficacy. Recent human complement-factor-B pharmacodynamic data strengthen the platform-level evidence but do not establish efficacy in RA or LN. The most informative early trials will therefore be biomarker-enriched and mechanism-rich, with prespecified criteria that distinguish target failure from delivery, dose, endotype, or safety failure.

## 1 Introduction

Autoimmune rheumatic diseases are heterogeneous disorders in which loss of immunological tolerance converges with tissue-specific inflammation, stromal activation, vascular injury, and progressive organ damage. Rheumatoid arthritis (RA), systemic lupus erythematosus (SLE), lupus nephritis (LN), and systemic sclerosis (SSc) illustrate complementary but biologically distinct frameworks, and the compartment that must be reached differs in each (1–6).

Many disease-relevant nodes are not readily addressed by extracellular pharmacology. Antibodies and receptor-fusion proteins are effective for accessible ligands or receptors, whereas small molecules can reach intracellular pathways but may be limited by selectivity, pathway redundancy, or systemic toxicity. Broad autoimmune-RNA reviews catalogue modalities and candidate targets (7), while recent arthritis-focused reviews emphasize siRNA nanocarriers and local nucleic-acid delivery (8, 9). The present review does not attempt another catalogue. Its added value is a disease- and compartment-specific decision framework that asks whether each program can connect human target biology to the intended compartment and cell, productive delivery, direct target engagement (TE), interpretable pharmacodynamics (PD), repeat-dose safety, and a realistic manufacturing path. This chain changes interpretation because a negative clinical result without delivery and target-engagement evidence cannot distinguish failure of target biology from failure of route, carrier, dose, endotype selection, or tolerability.

This review uses RA, SLE/LN, and SSc as deliberately selected and complementary frameworks rather than claiming comprehensive coverage of all autoimmune rheumatic diseases. It integrates modality, compartment and cell access, target engagement, PD visibility, repeat-dose organ and immune safety, manufacturing maturity, access considerations, and biomarker-guided patient selection. Sections 4 to 6 apply this framework to the distinct clinical and compartmental problems posed by each disease. The objective is to identify translationally coherent programs and the minimum evidence required for interpretable early clinical development (**Figure 1**).

**Figure 1:**
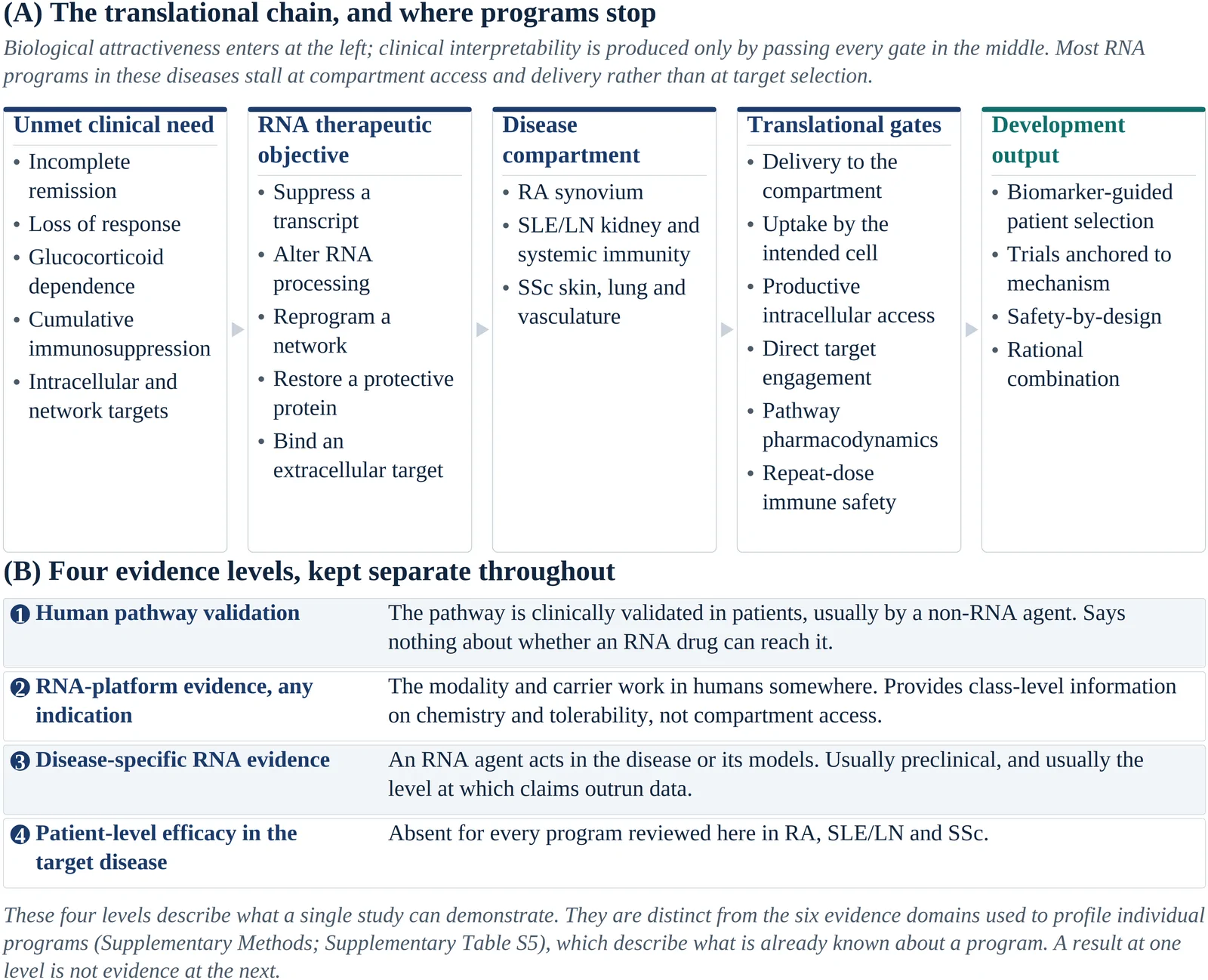
The framework applied throughout this review. **(A)** The sequence a program must complete, drawn as five stages with the requirements belonging to each; green marks the terminal stage. **(B)** The hierarchy of evidence levels, introduced under “Literature search and evidence classification” in Section 1.1 and applied to every program discussed.

### 1.1 Literature search and evidence classification

This narrative translational review used targeted, iterative literature searches rather than a Preferred Reporting Items for Systematic Reviews and Meta-Analyses (PRISMA)-based systematic-review process. PubMed/MEDLINE searches were updated through July 2, 2026, and were supplemented by ClinicalTrials.gov, regulatory-agency and professional-society documents, major rheumatology conference proceedings, sponsor disclosures, and reference-list screening. Search blocks combined RA, SLE/LN, or SSc terms with antisense oligonucleotide (ASO), small interfering RNA (siRNA)/RNA interference, microRNA (miRNA)/antagomiR/mimic, messenger RNA (mRNA), RNA aptamer, N-acetylgalactosamine (GalNAc), lipid nanoparticle (LNP), delivery, biodistribution, target engagement, PD, safety, and clinical-development terms. Documented PubMed/MEDLINE search blocks are provided in the **Supplementary Methods**. English-language peer-reviewed primary studies, contemporary guidelines, trial registries, and authoritative regulatory sources were prioritized. Conference abstracts and sponsor documents were used only when peer-reviewed reports were unavailable for recent program status or preliminary pharmacology and were not treated as evidence of patient efficacy. No meta-analysis or formal risk-of-bias instrument was applied. Final evidence inclusion and interpretation were reviewed by the authors.

The scope of this review was intentionally restricted to three complementary disease frameworks—RA, SLE/LN, and SSc—to permit sufficiently detailed, disease-specific evaluation of modality–delivery–biomarker alignment across synovial, systemic/renal immune-complex, and vascular–fibrotic compartments. Dermatomyositis and Sjögren disease were not included in the formal evidence synthesis because each would require a distinct compartment-specific search and translational appraisal beyond the defined scope of this review. Their exclusion should not be interpreted as indicating lower biological relevance or therapeutic potential for RNA-based approaches.

Evidence was separated at four levels: human pathway validation; RNA-platform evidence outside the target disease; disease-specific RNA evidence; and patient-level efficacy in the target disease. Each program was then profiled across six domains: (i) human disease-biology validation; (ii) disease-specific RNA evidence; (iii) delivery to the relevant compartment and cell population; (iv) human RNA target-engagement or PD evidence, explicitly labeled as in-disease or outside-disease; (v) patient-level efficacy; and (vi) repeat-dose safety and chemistry, manufacturing, and controls (CMC) maturity. Operational definitions, conservative rules for discordant or multifunctional studies, and source-hierarchy rules are provided in the **Supplementary Methods**. Assignments were discussed and agreed by the authors; this was a structured consensus appraisal rather than a validated score or formal duplicate risk-of-bias assessment. Near-term proof-of-mechanism (PoM) tractability denotes only the feasibility of an interpretable early human study and does not predict efficacy, approval, or active candidate status.

## 2 RNA therapeutic objectives and modality-selection logic

RNA therapeutics extend pharmacology to transcripts and to RNA-encoded protein expression (10), but platforms should be selected by therapeutic objective, molecular site of action, target compartment, intended cell, and measurable PD—not by sequence programmability alone. A liver-derived complement factor, a synovial macrophage transcript, a B-cell regulatory network, and a dermal fibroblast program present different delivery and safety problems. **Table 1** provides the comparative pharmacology.

**Table 1:** Comparative modality selection for RNA therapeutic objectives in rheumatology.

Therapeutic objective	Modality	Mechanism / site	Best-fit rheumatology use	Principal constraint	Key refs
Transcript degradation or RNA processing	ASO	RNase H1 degradation or steric splice/translation modulation; nucleus/cytoplasm	Complement, interferon, profibrotic or intracellular transcripts	Sequence/chemistry-specific renal, hepatic, platelet and hybridization toxicity	(11–16)
Defined transcript knockdown	siRNA	AGO2/RISC cleavage; cytoplasm	Liver-derived complement; cytokine, B-cell, myeloid or stromal targets	Extrahepatic delivery, endosomal escape, seed off-targets, innate sensing	(17–21, 24)
Network reprogramming	miRNA inhibitor or mimic	Suppresses or restores a post-transcriptional network	Macrophage, B-cell and fibroblast-state modulation	Pleiotropy and cell-context dependence; needs gene-set PD	(26–28)
Transient protein restoration	mRNA	Cytoplasmic translation of encoded protein	Protective, tolerogenic, antibody-expression or antifibrotic programs	Innate activation, non-target expression, carrier immunity and duration control	(25, 29–32)
Extracellular binding / targeting	RNA aptamer	High-affinity extracellular or cell-surface binding	Antagonist or targeting ligand for conjugates/nanoparticles	Nuclease stability, renal clearance, modification-related safety and sparse rheumatology evidence	(33–36)

Rows compare therapeutic objective, modality, mechanism, best-fit use, principal constraint, and key supporting references. ASO, antisense oligonucleotide; miRNA, microRNA; mRNA, messenger RNA; siRNA, small interfering RNA.

### 2.1 Transcript suppression and RNA processing: ASOs versus siRNAs

Antisense oligonucleotides (ASOs) are single-stranded molecules that can promote RNase H1-mediated degradation or sterically alter splicing, translation, and RNA–protein interactions. Their nuclear and cytoplasmic activity depends on gapmer or steric-blocking architecture, backbone chemistry, sugar modification, and intracellular localization (11–16). Small interfering RNAs (siRNAs) are double-stranded molecules that require cytosolic access, guide-strand loading into Argonaute 2 (AGO2)-containing RNA-induced silencing complex (RISC), and complementary mRNA cleavage; design must limit passenger-strand activity, seed-mediated off-target effects, and innate sensing (13, 17–21).

Both platforms can suppress a defined pathogenic transcript, but their translational fit differs. ASOs are preferable when splice modulation, nuclear RNA, or an RNase H1 mechanism is required. siRNAs are attractive for durable cytosolic knockdown, particularly when conjugate-mediated hepatocyte delivery is available. GalNAc conjugation has clinically mature hepatocyte delivery for liver-derived targets, whereas synovial macrophages, fibroblast-like synoviocytes (FLS), B cells, renal cells, dermal fibroblasts, and lung compartments still require less mature ligand-directed, LNP, biomimetic, or local systems (22–25). Candidate-specific renal, hepatic, platelet, hematologic, off-target, and immune monitoring is required for both classes; platform experience cannot substitute for sequence-, chemistry-, target-, and indication-specific assessment.

### 2.2 Network modulation: miRNA inhibitors and mimics

MicroRNAs regulate networks rather than single transcripts. Inhibitors may suppress pathogenic miRNAs, whereas mimics may restore protective programs across macrophage activation, B-cell hyperresponsiveness, fibroblast states, and matrix remodeling (26–28). Network leverage is also the principal risk: effects are pleiotropic and may differ across immune, stromal, endothelial, and repair compartments. Development therefore requires direct miRNA engagement, cell-aware gene-set PD, and broad assessment of unintended network effects.

### 2.3 Transient protein restoration or expression: mRNA

mRNA therapeutics introduce a coding sequence for transient cytoplasmic protein expression. Cap structure, untranslated regions, coding sequence, poly(A) tail, nucleoside composition, carrier, and route determine translation, duration, distribution, and innate activation (25, 29–32). The modality is best aligned with restoration of protective or immunoregulatory proteins, temporary tolerogenic signals, or local tissue modification. In chronic autoimmune disease, non-target expression, carrier reactogenicity, and repeat-dose immunity make local or cell-directed programs more tractable than nonspecific systemic expression.

### 2.4 Extracellular binding and targeting-enabled delivery: aptamers

Aptamers are structured single-stranded nucleic acids that bind extracellular proteins or cell-surface targets and may act as antagonists or targeting ligands. Chemical synthesis is reproducible and generally avoids cold-chain dependence, but unmodified RNA aptamers are vulnerable to nuclease degradation and rapid renal clearance. 2′-fluoro, 2′-O-methyl or other sugar modifications, terminal capping, circularization, multimerization, albumin-binding or lipid modules, and polyethylene glycol (PEG) conjugation (PEGylation) can improve stability or exposure, although modification may alter affinity and PEG can introduce hypersensitivity risk (33).

Rheumatology evidence remains preclinical: an IL-6 SOMAmer reduced arthritis in cynomolgus monkeys, and a chemically stabilized, cholesterol-modified DEK aptamer delivered by hydrogel microneedles attenuated collagen-induced arthritis in mice (34, 35). The broader REG1 experience is a cautionary clinical precedent: a phase III factor IXa RNA-aptamer program was terminated early because of severe allergic reactions associated with the current PEGylated formulation, and it did not improve ischemic outcomes (36). This was not an inflammatory-disease efficacy trial and does not establish an aptamer class effect; it illustrates that half-life extension and carrier chemistry are part of aptamer pharmacology. Comparative cost claims remain uncertain. Aptamers share solid-phase synthesis, purification, impurity control, and scale-up constraints with other synthetic oligonucleotides and are not inherently cheaper; short unconjugated sequences may be simpler than antibodies, whereas stabilization, conjugation, multimerization, PEGylation, or nanoparticle integration adds analytical and CMC burden. Aptamers are therefore treated here as an emerging binder/targeting class rather than a clinically mature rheumatology platform.

Across these modalities, the therapeutic objective should drive selection: transcript degradation favors ASO or siRNA; splice modulation favors steric-blocking ASO; hepatocyte-derived target suppression may favor GalNAc-conjugated oligonucleotides; network reprogramming may favor miRNA modulation; protein restoration favors mRNA; and extracellular antagonism or targeting-ligand functions may favor aptamers. The pharmacodynamic plan should be fixed before efficacy is interpreted; the disease-specific windows are set out in Section 7.1.

## 3 Delivery as the central translational gate in autoimmune rheumatology

Delivery is the principal translational gate for RNA therapeutics in autoimmune rheumatology. A candidate must survive administration, reach the relevant compartment, enter the intended cell population, access the appropriate intracellular site, engage its target, produce measurable PD, and remain tolerable with repeated dosing (22, 23, 37–40). This is particularly demanding for extrahepatic, inflamed, vascularly abnormal, fibrotic, or cellularly heterogeneous tissues.

### 3.1 From systemic exposure to compartment pharmacology

Systemic exposure does not establish compartment pharmacology. Nuclease degradation, renal clearance, serum protein binding, reticuloendothelial uptake, hepatic or splenic tropism, endothelial barriers, and variable tissue penetration can separate plasma exposure from biologically meaningful exposure in synovium, kidney, skin, lung, lymphoid tissue, vasculature, or tissue-resident cells (15, 22, 40).

Compartment barriers are disease-specific. In RA, vascular permeability may permit nanoparticle entry without productive delivery to macrophages, FLS, or deeper stromal compartments because synovial turnover, lymphatic clearance, matrix organization, phagocytic sequestration, and receptor heterogeneity remain limiting. In SLE/LN, systemic immune, hepatocyte-directed, and renal-cell delivery must be distinguished: suppressing a circulating or liver-derived mediator may influence nephritis without proving kidney-cell exposure. In SSc, vascular injury and fibrotic matrix constrain access; serial skin sampling can support early PD, whereas lung programs require biodistribution, pulmonary tolerability, and repeat-dose evidence.

Cellular uptake is not equivalent to pharmacological activity. Nanoparticles may be internalized yet remain in endosomes or lysosomes. Productive cytosolic access is required for siRNA and mRNA, whereas ASOs may require nuclear and/or cytoplasmic access according to mechanism. Endosomal entrapment and recycling can determine potency, dose, off-target exposure, immune activation, and therapeutic index (41–43). **Figure 2** summarizes the resulting delivery cascade.

**Figure 2:**
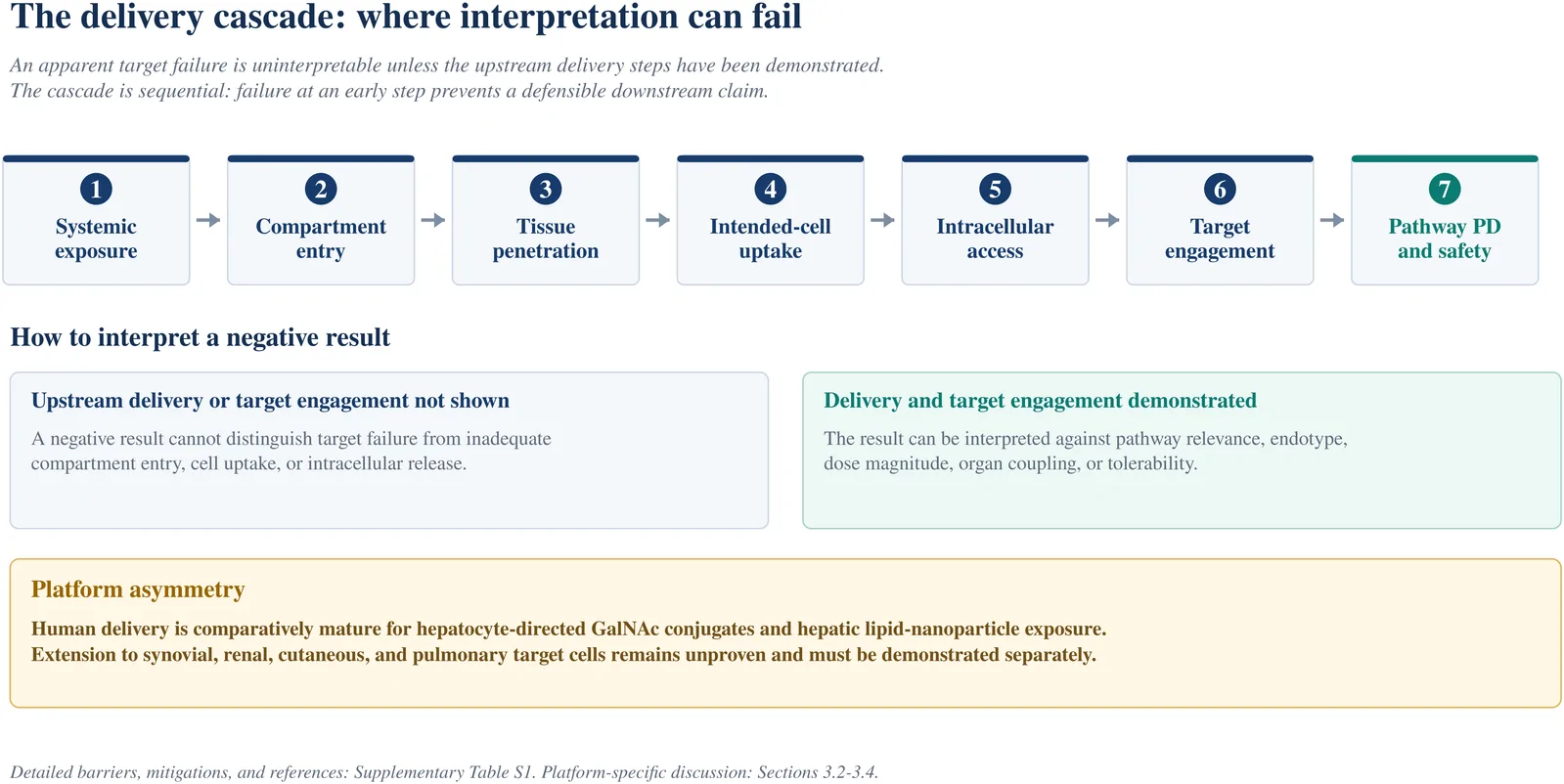
The delivery cascade and its interpretive consequences. The seven delivery and pharmacology requirements are shown as a high-level cascade. The two interpretive paths distinguish a result obtained before upstream delivery or target engagement is established from one obtained after those requirements are demonstrated. The highlighted statement records the platform asymmetry that governs the remainder of the review. Detailed barriers, mitigations, and references are provided in **Supplementary Table S1**.

### 3.2 Systemic platforms: GalNAc conjugates and LNPs

GalNAc conjugation is the most mature systemic oligonucleotide-delivery strategy for hepatocytes and is best suited to predominantly liver-derived targets (24, 37, 38). Hepatic knockdown does not demonstrate delivery to synovium, kidney cells, skin, lung, or immune cells; autoimmune programs should link target suppression to organ-specific biomarkers and outcomes.

LNP performance depends on ionizable-lipid chemistry, size, helper lipids, cholesterol, polyethylene glycol (PEG)-lipid content, pKa, surface properties, and formulation stability (25, 32, 39, 44, 45), while systemic distribution often remains liver- or spleen-biased. Animal organ tropism should not be equated with human cell delivery; translation requires functional target engagement, immune-safety assessment, CMC reproducibility, and repeat-dose tolerability.

### 3.3 Extrahepatic and local delivery strategies

Extrahepatic delivery remains the major barrier for many autoimmune programs. Targeted nanoparticles, biomimetic or ligand-directed carriers, depots, and intradermal or intra-articular systems may increase compartment exposure but must be judged by functional target engagement, not uptake alone.

In RA, abundant phagocytic synovial macrophages may be more tractable targets than FLS and can be targeted through phagocytosis, scavenger or mannose receptors, folate-receptor biology, inflammatory homing, or biomimetic membranes (46–48). Yet macrophage sequestration may divert particles from FLS and deeper stroma; biopsy-based cell-resolved PD, imaging, synovial-fluid markers, and ex vivo human tissue models are needed to distinguish productive delivery from accumulation.

Local or intra-articular administration can increase joint exposure and reduce systemic distribution but remains limited by synovial-fluid clearance, lymphatic drainage, macrophage uptake, and endosomal trapping. Its most credible roles are PoM, adjunctive treatment, or a dominant-compartment strategy, not replacement of systemic control in polyarticular disease.

Skin-directed delivery offers accessible PoM in SSc, whereas lung programs require biodistribution and safety evidence. In SLE/LN, systemic immune or liver-derived targets are nearer-term than direct renal-cell delivery.

### 3.4 Immune safety and repeat-dose tolerability

Immune safety is part of delivery pharmacology. Depending on sequence, chemistry, dose, route, and formulation, therapeutic RNAs and carriers may activate endosomal Toll-like receptors, cytosolic RNA sensors, interferon programs, inflammatory cytokines, complement, or anti-carrier responses (18, 19, 31, 49–52). The risk is amplified in SLE/LN, where nucleic-acid sensing and complement are integral to disease biology.

Chemical modification reduces but does not guarantee immune silence (31). Candidate-specific testing should use relevant human immune-cell systems and include interferon-stimulated genes, cytokines, complement markers, and disease-appropriate cellular readouts. RA programs additionally require infection and myeloid/synovial monitoring; SSc programs require vascular and pulmonary tolerability and assessment of unintended profibrotic or inflammatory shifts.

Repeat dosing may alter biodistribution, accelerate clearance, reduce PD, or increase hypersensitivity through anti-PEG, anti-lipid, or other anti-carrier responses (49–52). LNP, liposomal, polymeric, biomimetic, and depot systems therefore require longitudinal pharmacokinetic/pharmacodynamic (PK/PD), complement, hypersensitivity, organ-toxicity, and local-tolerability evaluation.

Accordingly, delivery selection begins with the causal source and intended cell: hepatocyte-directed conjugates for liver-derived targets; validated local or cell-directed systems for synovial, immune, dermal, renal-cell, or pulmonary targets. Advancement requires a linked exposure–target-engagement–PD–safety dataset rather than organ uptake alone.

## 4 RA: RNA strategies at the synovial immune–stromal interface

### 4.1 The unmet clinical problem

Despite effective biologic and targeted synthetic therapy, a substantial proportion of patients with RA do not achieve sustained remission, remain glucocorticoid-dependent, or accumulate infection and toxicity risk from continued systemic immunosuppression (1, 2). The problem an RNA program has to solve is therefore not the absence of an effective drug class, but the absence of a way to act on targets that extracellular agents cannot reach.

RA is sustained by interactions among synovial macrophages, FLS, T and B cells, plasma cells, dendritic and endothelial cells, and osteoclast-lineage cells. Their cytokines, chemokines, proteases, matrix-remodeling enzymes, immune complexes, complement products, and osteoclastogenic mediators drive synovitis, pannus, cartilage destruction, and bone erosion (1, 2, 53–55).

Single-cell and tissue studies demonstrate multiple macrophage, fibroblast, lymphocyte, endothelial, and stromal states rather than a uniform RA synovium (53–56). Biopsy-driven studies also associate tissue phenotypes with differential response to biologics (57–59). An RNA target may therefore be dominant in one endotype but redundant in another.

### 4.2 What an RNA program would have to add

RNA therapeutics are most distinctive when they address intracellular, stromal, transcriptional, or network-level biology not readily controlled by extracellular agents. Candidate axes include nuclear factor kappa B (NF-κB) signaling, Bruton’s tyrosine kinase (BTK)-associated B-cell or myeloid activation, Notch signaling, macrophage state, FLS invasiveness, miRNA programs, complement amplification, and protective-pathway restoration. Pathway validity must still be separated from delivery and clinical advantage over established therapies.

### 4.3 Compartment, cell population, and route

The two direct synovial propositions are considered first, followed by liver-derived targets that act on the joint indirectly. Filled segments summarize how far delivery to the named population has been demonstrated. Delivery, not target choice, is the step that most often determines whether an RA result can be interpreted at all (**Figure 3**).

**Figure 3:**
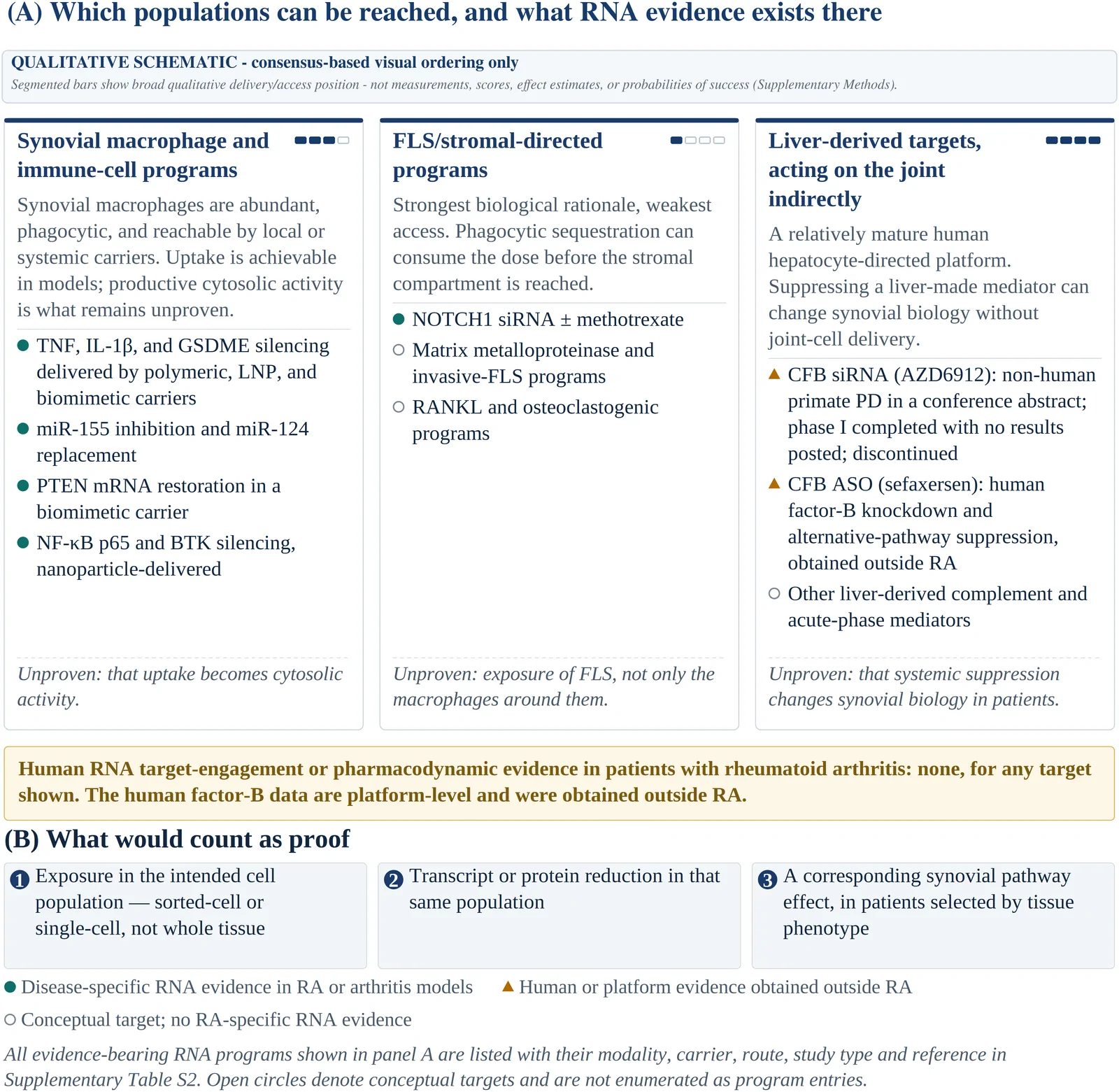
Rheumatoid arthritis: which populations can be reached, and what evidence exists for each. **(A)** Three propositions are arranged to follow Section 4.3 rather than evidence strength and therefore place the hepatocyte-directed platform last. The segmented bars are consensus-based qualitative schematic cues for broad delivery/access position; they are not measurements, scores, effect estimates, or probabilities of success. A single filled segment indicates that direct delivery to the named population has not been demonstrated. The highlighted statement records the central negative finding of this section. **(B)** The observations that would convert a future result in this disease into evidence about the target rather than about the platform.

#### 4.3.1 Synovial macrophage and immune-cell programs

Most RA-focused RNA evidence remains preclinical. Tumor necrosis factor (TNF) siRNA delivered by polymerized siRNA/thiolated glycol-chitosan nanoparticles or LNP formulations reduced experimental arthritis (60, 61). These studies establish biological and delivery proof of concept, not superiority to anti-TNF biologics. A clinically persuasive program would require a clear advantage such as local exposure, reduced systemic immunosuppression, durable but reversible knockdown, endotype selection, or rational combination.

Lipidoid–polymer nanoparticles delivering interleukin-1 beta (IL-1β) siRNA reduced disease activity in a macrophage-biased mouse arthritis model (62). The result supports myeloid-directed cytokine knockdown, but IL-1β is not a dominant broad RA clinical axis; translation would likely require a selected myeloid-inflammatory phenotype or a local pathway-amplification objective.

A 2026 biomimetic hybrid system co-delivered Gasdermin E (GSDME) siRNA and 4-octyl itaconate to folate-receptor-positive inflammatory macrophages, coupling inhibition of pyroptosis with metabolic reprogramming (63). The study strengthens macrophage-targeted combination logic but does not establish single-agent GSDME-siRNA efficacy; attribution, component interaction, manufacturing complexity, and repeat-dose immune safety must be resolved.

miR-155 is increased in RA synovium and synovial-fluid macrophages and acts as a pro-inflammatory regulator in clinical and experimental arthritis (64). Inhibition is therefore a macrophage-reprogramming concept, but systemic exposure could affect myeloid, B-cell, T-cell, regulatory, and host-defense programs. Cell-directed delivery and gene-set-level immune monitoring are required.

Restoration of miR-124 represents the converse strategy. Methotrexate-conjugated hybrid micelles co-loaded with a miR-124 mimic showed activity in experimental RA (65). Because the platform contains two active components, translation requires dose attribution, reproducible delivery, and confirmation of network effects in human synovial tissue.

A biomimetic macrophage-membrane nanoparticle has also combined TNF-receptor decoy activity on the engineered carrier with delivery of phosphatase and tensin homolog (PTEN) mRNA to inflamed joints (66). The whole platform reduced inflammatory signaling and joint injury in preclinical RA, but efficacy cannot be attributed to PTEN restoration alone. Translation will require component-level controls, localization and duration of encoded PTEN protein, innate and carrier immunity, non-target expression, and repeat-dose assessment.

Peptide–siRNA nanocomplexes targeting the NF-κB p65 subunit suppressed experimental arthritis and reduced local cytokine expression, while hybrid systems have combined p65 siRNA with methotrexate (67, 68). Because NF-κB is essential for host defense and cell survival, local or cell-directed delivery and direct pathway PD are prerequisites.

Nanoparticle-delivered BTK siRNA reduced experimental arthritis (69). The target is expressed in B-lineage and myeloid cells rather than in fibroblast-like synoviocytes, so this is an immune-cell rather than a stromal program, and cell specificity, pathway redundancy and infection risk remain the operative limitations.

#### 4.3.2 FLS/stromal-directed programs

NOTCH1-targeting siRNA nanoparticles, alone or combined with methotrexate, reduced inflammation and structural damage in collagen-induced arthritis (70, 71). These studies support stromal targeting, but efficient delivery to the FLS compartment rather than to the macrophages that surround it, and clinically interpretable pathway modulation, remain unproven.

#### 4.3.3 Liver-derived targets acting on the joint indirectly

Complement can amplify synovial inflammation at the interface of autoantibodies, immune complexes, innate immunity, and tissue injury (72). Because selected complement proteins are largely liver-derived, systemic oligonucleotide suppression is more tractable than direct synovial complement delivery. Any GalNAc-based strategy should therefore be described as hepatic pathway modulation, not synovial RNA delivery.

Complement factor B (CFB) is a central alternative-pathway amplification protein. AZD6912, a GalNAc-conjugated siRNA targeting CFB mRNA, produced hepatic transcript knockdown, circulating factor B reduction, and alternative-pathway PD in non-human primates reported in a 2024 American College of Rheumatology (ACR) abstract (73). ClinicalTrials.gov lists a completed randomized single-ascending-dose Phase I study in 40 healthy participants (NCT06115967), with no results posted (74).

AstraZeneca subsequently reported discontinuation of AZD6912 for strategic portfolio prioritization (75). The candidate is therefore not an active RA program. Class-level human target engagement is nevertheless available from sefaxersen, a GalNAc-conjugated CFB ASO: randomized phase I studies in 54 healthy participants reported up to 69% mean factor-B reduction after repeated dosing, parallel Bb and alternative-pathway suppression, and no identified safety signal in those studies (76). A subsequent single-arm phase II study of the same agent in 23 patients with IgA nephropathy reported approximately 70% reduction in plasma factor B, selective alternative-pathway suppression without a comparable classical-pathway effect, and a 43% reduction in 24-hour urinary protein excretion at week 29 with stable estimated glomerular filtration rate (eGFR), alongside transient and reversible transaminase elevations in three participants (77). Together these data demonstrate human systemic CFB suppression after hepatocyte-directed dosing, with alternative-pathway pharmacodynamics and, in a non-rheumatological glomerular disease, a linked organ-level readout; they do not establish synovial target engagement or RA efficacy.

### 4.4 Translational appraisal and minimum evidence requirements

Three things must be shown before an RA result can be read as evidence about the target rather than about the platform: exposure in the intended cell population rather than in whole tissue; transcript or protein reduction in that same population; and a corresponding synovial pathway effect in patients selected by tissue phenotype.

Future complement-directed RA studies should enrich for complement-active biology rather than unselected disease. Candidate markers include split products, functional pathway assays, circulating or synovial complement signatures, autoantibody status, and tissue evidence of complement-amplified inflammation. The decisive question is whether systemic factor B suppression changes synovial biology and clinical disease without unacceptable infection risk.

RA target prioritization should distinguish pathway validity from program readiness. Cytokine silencing must outperform or complement effective biologics; NF-κB, BTK, and Notch strategies require local or cell-directed delivery; miRNA and combination platforms require attribution and network-level PD; and systemic complement suppression requires biomarker enrichment and proof of synovial relevance.

On current evidence, the most likely reason for an RA RNA program to fail is not that the target is wrong. For macrophage-directed programs, bulk-tissue uptake or knockdown may be reported without cell-resolved proof of productive activity in the intended human synovial population, so a negative result cannot separate an inactive target from an undelivered one. For FLS and stromal programs it is that the dose is consumed by phagocytic cells before the intended compartment is reached. For liver-directed complement programs it is that systemic pathway suppression is achieved and never linked to synovial biology in patients selected for complement amplification. A fourth constraint is specific to RA rather than to RNA: a program that clears all three steps must still demonstrate an advantage over effective biologic and targeted synthetic therapy, whether in local exposure, reduced systemic immunosuppression, durability, endotype selection, or combination logic. **Supplementary Table S2** provides the program-level evidence profile.

## 5 SLE and LN: RNA strategies for interferon, B-cell, co-stimulation, and complement axes

### 5.1 The unmet clinical problem

SLE is driven by interacting defects in nucleic-acid handling, innate sensing, type I interferon activity, autoreactive B-cell responses, T-cell help, immune complexes, complement, and tissue injury (3, 4, 78, 79). LN adds intrarenal inflammation, vascular injury, and chronic scarring that can produce irreversible kidney damage (78).

Modern SLE/LN care emphasizes early control, hydroxychloroquine when appropriate, glucocorticoid minimization, rational immunosuppressive combinations, biologic or targeted add-on therapy, kidney protection, and organ-specific monitoring (80–84). Relapsing nephritis, refractory extrarenal disease, cumulative immunosuppression, infection, and renal progression remain important unmet needs.

### 5.2 What an RNA program would have to add

RNA strategies can access interferon-regulatory, nucleic-acid-sensing, B-cell survival, co-stimulatory, complement, and miRNA-regulated networks. Human efficacy of anifrolumab and belimumab validates the interferon-receptor and B-cell activating factor (BAFF; also known as B lymphocyte stimulator [BLyS]) pathways, respectively, but does not establish RNA feasibility (85, 86). Disease-relevant RNA examples now include interferon regulatory factor 5 (IRF5)/BLyS–BAFF silencing, anti-BAFF mRNA delivery, cluster of differentiation 40 (CD40) silencing, factor B ASO, and B-cell-targeted anti-miR-7 (87–91).

### 5.3 Compartment, cell population, and route

Four delivery propositions are routinely conflated in SLE and LN and are separated here: suppression of a liver-derived mediator, delivery to circulating and systemic immune cells, cell-directed delivery to B cells, and delivery to glomerular, tubular, or kidney-resident immune cells. They are ordered below by how far each has actually been demonstrated (**Figure 4**).

**Figure 4:**
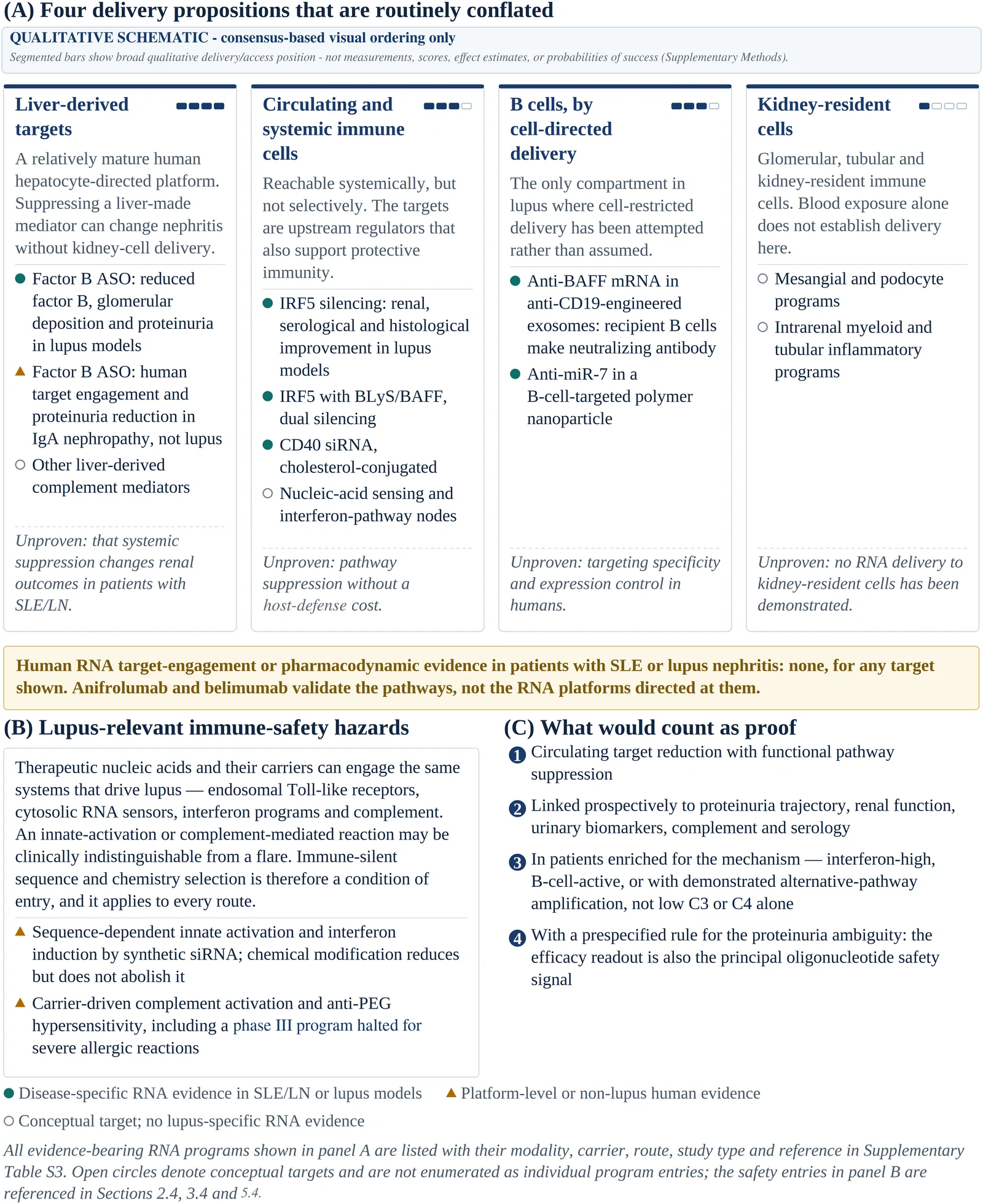
Systemic lupus erythematosus and lupus nephritis: four propositions that are commonly treated as one. **(A)** Four delivery arguments presented as a qualitative narrative of demonstrated access; the second and third share the same broad category, and their sequence follows Section 5.3. The segmented bars are consensus-based qualitative schematic cues for broad delivery/access position, not measurements or scores. A single filled segment indicates that direct delivery to the named population has not been demonstrated, and the factor B antisense entry is the agent named sefaxersen in **Figure 3**. The highlighted statement records the central negative finding of this section. **(B)** Hazards particular to lupus. **(C)** The observations that would make a future result interpretable.

#### 5.3.1 Liver-derived targets

Complement has context-dependent protective and pathogenic roles in lupus. Deficiency of early classical-pathway components predisposes to disease, whereas complement activation and deposition contribute to immune-complex injury. Development should therefore be pathway-, compartment-, stage-, and subgroup-specific rather than based on global “complement blockade.”

Selected complement proteins are predominantly liver-derived and accessible to mature hepatocyte-directed oligonucleotide platforms (24, 37, 38, 90). Sefaxersen trials demonstrate circulating factor B and alternative-pathway suppression after GalNAc-ASO dosing in healthy participants (76) and sustained factor B suppression in IgA nephropathy, where proteinuria fell and eGFR remained stable (77). These data establish systemic delivery and PD outside LN, not direct renal-cell delivery or efficacy in LN.

ASO-mediated inhibition of CFB reduced factor B, glomerular complement deposition, proteinuria, renal pathology, and mortality in lupus models (90). This establishes disease-specific preclinical linkage between systemic alternative-pathway modulation and renal injury, not human LN efficacy.

#### 5.3.2 Circulating and systemic immune cells

Type I interferon drives dendritic-cell maturation, B-cell activation, autoantibody production, and tissue inflammation in SLE (4). IRF5 is an RNA-addressable upstream regulator supported by SLE genetics and functional biology (79). In lupus-prone models, silencing IRF5 alone or together with BLyS/BAFF improved renal, serological, and histological outcomes (87).

Upstream control is attractive but could impair protective innate immunity. *IRF5*-directed development should therefore prioritize interferon-high biology and measure interferon-stimulated gene scores, cytokine and myeloid activation, autoantibodies, complement, urinary inflammatory markers, proteinuria, renal function, and biopsy context rather than relying on a single blood marker.

Autoreactive B-cell survival, class switching, plasmablast differentiation, autoantibody production, antigen presentation, and immune-complex formation are central to SLE/LN. BAFF is clinically validated by belimumab in active LN (86), so an RNA strategy must offer a defined advantage in distribution, durability, combination logic, treatment burden, or biomarker-selected use.

Dual IRF5 and BLyS/BAFF silencing improved experimental LN (87), illustrating coordinated control of reinforcing innate and B-cell pathways. The combination also increases dose attribution, pathway-confirmation, host-defense, and patient-selection complexity.

CD40–CD40 ligand (CD40L) signaling connects T-cell help, B-cell and antigen-presenting-cell activation, autoantibody responses, and renal inflammation. Cholesterol-conjugated anti-CD40 siRNA reduced proteinuria, anti-DNA antibodies, and renal pathology in experimental LN (89). Because CD40 supports protective adaptive immunity, systemic suppression requires infection monitoring and a stronger case for immune- or renal-compartment delivery.

#### 5.3.3 B cells, by cell-directed delivery

Cluster of differentiation 19 (CD19)-targeted exosomes have delivered anti-BAFF mRNA to B cells in lupus mice, enabling the recipient cells to produce BAFF-neutralizing antibody and reducing renal injury and disease features (88). This is a preclinical targeted-mRNA platform, not human efficacy. Translation must address exosome manufacturing, dose and expression control, anti-CD19 targeting specificity, anti-BAFF antibody kinetics, off-target transfection, and repeat-dose immunogenicity.

B-cell-targeted polymer nanoparticles delivering anti-miR-7 reduced B-cell activation, anti-double-stranded-DNA antibodies, and lupus-like disease features in mice (91). The result supports network modulation within a defined cell type; direct miRNA engagement, gene-set PD, and broad assessment of pleiotropic effects remain essential.

#### 5.3.4 Kidney-resident cells

We identified no demonstrated RNA delivery to glomerular, tubular, or kidney-resident immune cells in lupus nephritis. Mesangial, podocyte, and intrarenal myeloid programs remain conceptual targets, and blood exposure does not establish renal-compartment delivery.

### 5.4 Translational appraisal and minimum evidence requirements

Every program should prespecify the disease compartment, modality, route, delivery hypothesis, direct target-engagement assay, renal or systemic PD, enrichment strategy, and immune-safety plan. In LN, systemic pathway effects must be connected to proteinuria trajectory, renal function, urinary biomarkers, complement activity, and biopsy-informed context when clinically appropriate (78, 81, 83, 84).

Patient selection should integrate complement split products or functional alternative-pathway assays with renal activity, urinary markers, serology, and biopsy context where appropriate (78, 81, 83, 84, 92). Low C3 or C4 alone does not establish factor B-dependent amplification.

Circulating factor B reduction is target engagement, not renal benefit. Confirmation requires prospective linkage to pathway suppression, proteinuria, renal function, urinary biomarkers, and biopsy context; safety monitoring should cover infection, complement suppression, and carrier-related complement activation.

SLE/LN development should prioritize immune safety, direct PD, and biomarker enrichment. Interferon programs require interferon-signature and innate-activation readouts; B-cell programs require subsets, plasmablasts, autoantibodies, BAFF, and immune-complex markers; complement programs must pair systemic target suppression with renal measures; and miRNA programs require cell-type and gene-set PD.

Near-term opportunities favor measurable systemic PD for liver-derived complement factors and may favor selected circulating immune pathways over direct renal-cell delivery, provided human immune-cell access is demonstrated.

Against this background, SLE is a particularly high-risk setting for therapeutic nucleic acids because unintended Toll-like receptor or cytosolic RNA sensing, interferon induction, inflammatory cytokines, complement activation, or immune-complex-like reactions could reinforce disease (18, 19, 31, 49). This paradox makes candidate-specific immune-silencing chemistry, carrier selection, and human immune-cell testing central rather than ancillary.

This is the failure mode that most distinguishes SLE/LN from the other two diseases. An innate-activation or complement-mediated reaction may be clinically indistinguishable from a flare, so immune-silent sequence and chemistry selection is a condition of entry rather than late safety work, and it applies to every route, including hepatocyte-directed conjugates. The second failure mode is interpretive: circulating target reduction is readily achieved and readily over-read, and without a prospectively specified link to proteinuria trajectory, renal function, urinary biomarkers, and complement measures it cannot be distinguished from a systemic effect with no renal consequence. **Supplementary Table S3** provides the program-level evidence profile for SLE/LN.

## 6 SSc: RNA therapeutics at the autoimmune–vascular–fibrotic interface

### 6.1 The unmet clinical problem

SSc combines immune dysregulation, microvascular injury, fibroblast activation, extracellular-matrix accumulation, and progressive skin and internal-organ fibrosis (5, 6). Its transcript- and network-driven biology makes it relevant to RNA therapeutics, but each compartment requires separate delivery and endpoint logic.

Organ-based therapy has advanced, particularly for skin disease, interstitial lung disease (ILD), and selected inflammatory or vascular manifestations, with mycophenolate, nintedanib, rituximab, and tocilizumab used in defined contexts (93–96). Established fibrosis remains difficult to reverse, and progressive skin, lung, vascular, gastrointestinal, cardiac, or renal disease continues despite treatment.

### 6.2 What an RNA program would have to add

RNA opportunities include transforming growth factor beta (TGF-β)-responsive and matrix-remodeling programs, cellular communication network factor 2/connective tissue growth factor (CCN2/CTGF), fibroblast-state regulation, antifibrotic miRNAs, and protective-protein restoration. Serial skin biopsy provides an accessible PD window, but organ-specific efficacy requires separate evidence.

Activated fibroblasts and myofibroblasts show increased matrix production, contractility, altered growth-factor responses, impaired resolution, and reciprocal signaling with immune and vascular cells. These features create RNA-addressable targets at the transcript, post-transcriptional, and protein-restoration levels, while also creating a dense matrix barrier to delivery.

Candidate approaches include ASO or siRNA suppression of profibrotic transcripts, miRNA replacement or inhibition, and mRNA restoration of protective regulators. The translational bar is high: active fibroblasts must be reached in skin or lung, pathway redundancy must be addressed, and interference with vascular homeostasis, wound healing, host defense, and tissue repair must be excluded.

### 6.3 Compartment, cell population, and route

SSc differs from RA and SLE/LN in one respect that shapes every program: one compartment is directly and repeatedly sampleable, and the compartments driving most mortality are not comparably accessible. The four are ordered below by how far delivery to each compartment has been demonstrated; pharmacodynamic accessibility is treated separately (**Figure 5**).

**Figure 5:**
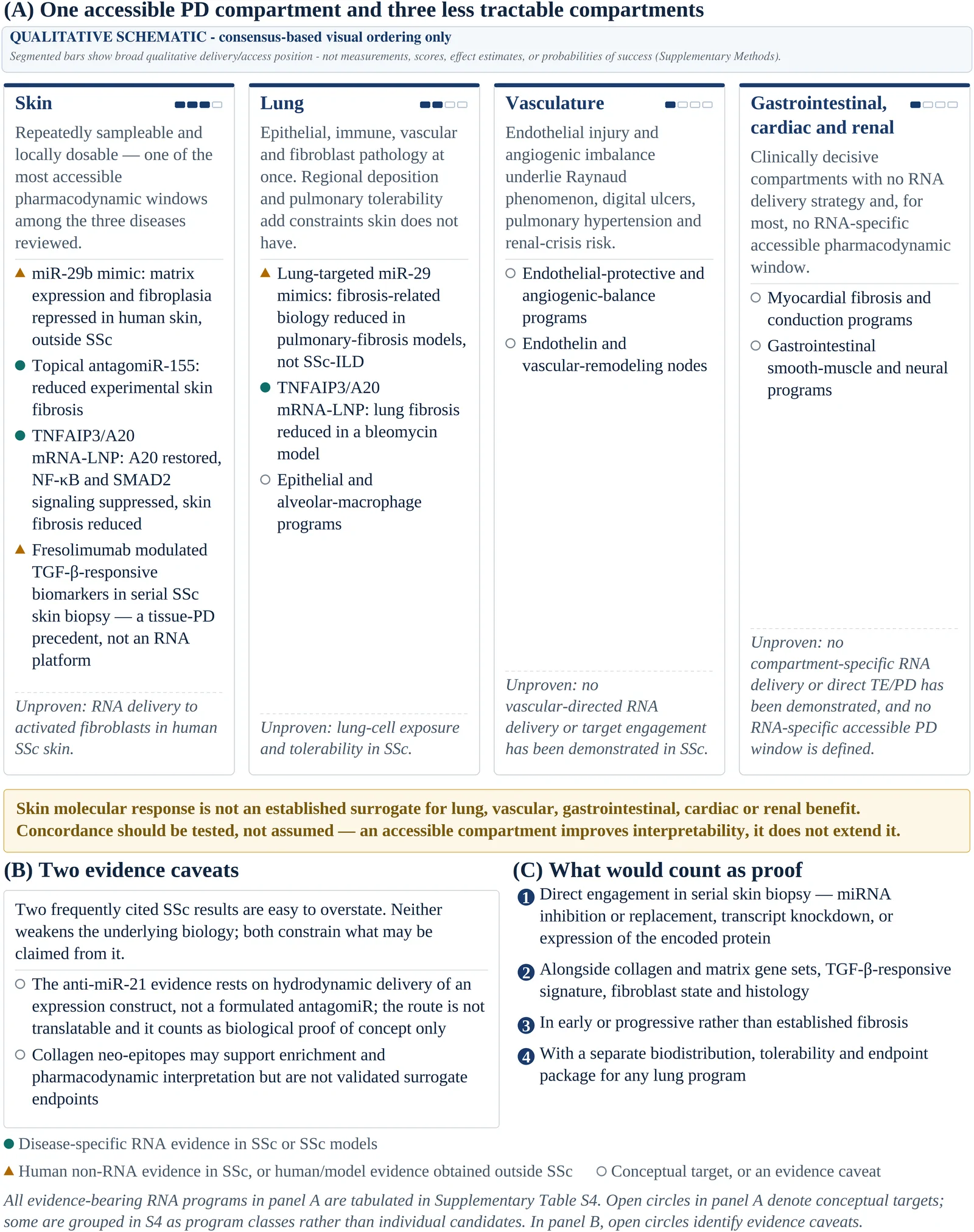
Systemic sclerosis: one accessible PD compartment and three less tractable compartments. **(A)** Four compartments presented as a qualitative narrative of demonstrated access; the last two share the same broad category. The segmented bars are consensus-based qualitative schematic cues for broad delivery/access position, not measurements or scores. A single filled segment indicates a compartment for which direct access has not been shown. The A20 messenger RNA program appears in both the skin and the lung card because it has been examined in a model of each. The highlighted statement records the constraint that limits the value of the accessible compartment. **(B)** Two caveats attaching to frequently cited results; the anti-miR-21 caveat concerns a result not listed in panel **(A)**. **(C)** The observations that would make a future result interpretable.

#### 6.3.1 Skin

Serial biopsy makes skin the most accessible PD compartment and can quantify miRNA or transcript engagement, encoded protein, matrix and TGF-β-responsive signatures, fibroblast states, and histology. The modified Rodnan skin score remains clinically important, but molecular target engagement should precede claims of disease modification.

A TGF-β-responsive signature is associated with a subset of diffuse cutaneous SSc and greater severity (97). Skin transcriptomics identify inflammatory and fibroproliferative subsets, and single-cell studies resolve fibroblast heterogeneity, including secreted frizzled-related protein 2 (SFRP2)-high/dipeptidyl peptidase 4 (DPP4)-expressing progenitor populations implicated in myofibroblast development (98–100). These data support molecular enrichment rather than one-size-fits-all development.

Fresolimumab is not an RNA therapeutic, but its small open-label SSc study showed rapid modulation of TGF-β-regulated biomarkers in serial skin biopsies (101). It therefore provides a human tissue-PD precedent, not validation of an RNA platform or a surrogate relationship between skin biomarkers and internal-organ benefit.

The miR-29 family negatively regulates multiple extracellular-matrix transcripts and is reduced in SSc fibroblasts and skin (102). Replacement may therefore suppress a coordinated fibrotic program rather than one collagen transcript.

Remlarsen, a miR-29b mimic, repressed matrix expression and fibroplasia in human skin wound models and human proof-of-mechanism studies outside SSc (103). Lung-targeted miR-29 mimics reduced fibrosis-related biology in pulmonary-fibrosis models and lung-relevant systems (104, 105). These data support cutaneous and pulmonary platform logic, but not patient efficacy in SSc or SSc-ILD.

Collagen neo-epitopes may reflect matrix remodeling and progression risk and could support enrichment or PD interpretation but are not validated surrogate endpoints (106). In a murine bleomycin model, hydrodynamic administration of an anti-miR-21 expression plasmid/miRZip construct reduced skin and lung fibrosis and signal transducer and activator of transcription 3 (STAT3)-associated inflammation (107); it should not be described as a formulated antagomiR. Topical antagomiR-155 also reduced experimental skin fibrosis (108). Both miRNAs are pleiotropic across fibroblast, immune, endothelial, and repair programs, making route and cell context decisive.

A recent preclinical study delivered tumor necrosis factor alpha-induced protein 3 (TNFAIP3; A20) mRNA in LNPs to human dermal fibroblasts and by weekly intramuscular dosing in a bleomycin SSc model (109). The platform restored A20 protein, suppressed tumor necrosis factor receptor-associated factor 6 (TRAF6)/NF-κB and downstream regulatory element antagonist modulator (DREAM)–SMAD family member 2 (SMAD2) signaling, and reduced skin and lung fibrosis. These findings support protective-pathway restoration, but do not establish fibroblast-specific delivery, human tissue exposure, chronic tolerability, or patient efficacy.

#### 6.3.2 Lung

SSc-ILD is a more difficult compartment involving epithelial, immune, vascular, and fibroblast pathology. Programs should demonstrate lung-relevant biodistribution and cell exposure, target engagement, pulmonary tolerability, repeat-dose safety, and linkage to forced vital capacity, diffusing capacity, high-resolution computed tomography, or appropriately qualified matrix biomarkers. Skin response cannot substitute for lung pharmacology.

#### 6.3.3 Vasculature

Vascular-directed RNA concepts remain early. Endothelial injury and angiogenic imbalance contribute to Raynaud phenomenon, digital ulcers, pulmonary hypertension, renal-crisis risk, and ischemia. Such programs require tissue-specific delivery and close monitoring of blood pressure, renal function, vascular events, wound healing, and non-target expression.

#### 6.3.4 Gastrointestinal, cardiac, and renal compartments

We identified no RNA delivery strategy for these compartments in SSc, and for most, no RNA-specific tissue-level pharmacodynamic window has been defined. They are clinically decisive and translationally unaddressed and should be described as such rather than folded into a general antifibrotic claim.

### 6.4 Translational appraisal and minimum evidence requirements

The minimum evidence for a skin-directed SSc program is direct engagement in serial skin biopsy—miRNA inhibition or replacement, transcript knockdown, or expression of the encoded protein—read alongside collagen and matrix gene sets, TGF-β-responsive signatures, fibroblast state, and histology, in early or progressive rather than established fibrosis. Any lung program requires its own biodistribution, tolerability, and endpoint package; it does not inherit the skin package.

Where target biology and route support a skin-first strategy, a staged program should establish delivery, target engagement, and antifibrotic molecular effects in an accessible compartment before expansion with organ-specific biodistribution, durability, safety, and endpoints.

Despite the tractability of this staged strategy, no RNA therapeutic is an established SSc treatment. The fibrosis biology is strong and the skin PD window is accessible, but most evidence remains preclinical, derives from related fibrosis systems, or provides human cutaneous proof of mechanism outside SSc.

An SSc program is therefore more likely to fail in interpretation than in biology. The skin compartment makes early evidence readable but cannot support claims of internal-organ benefit: skin molecular response is not an established surrogate for lung, vascular, gastrointestinal, cardiac, or renal outcomes, and concordance should be tested rather than assumed. Attribution is a second failure mode because frequently cited SSc results derive from combination platforms, non-translatable delivery routes, or fibrosis systems outside SSc, so a positive early result may belong to the carrier, route, or model rather than the RNA. **Supplementary Table S4** summarizes current opportunities.

## 7 Pharmacology, immune safety, and early clinical trial design

Early clinical pharmacology should link route and exposure to compartmental delivery, target engagement, pathway PD, organ response, and repeat-dose safety.

### 7.1 Target engagement and pharmacodynamic hierarchy

A five-layer hierarchy should assess: (1) tissue and cell exposure; (2) direct target engagement, including transcript/protein reduction, splice change, miRNA modulation, or encoded-protein expression; (3) pathway PD; (4) tissue or organ response; and (5) clinical benefit. Prespecified go/no-go criteria across these layers distinguish delivery, target, dose, endotype, and timing failures.

Disease-specific windows should anchor this hierarchy. RA programs may require synovial biopsy or fluid, imaging, and cell-aware transcriptomics; liver-directed RA complement programs must connect systemic factor B PD to synovitis. SLE/LN programs may combine interferon scores, B-cell subsets, serology, complement, urinary biomarkers, proteinuria, renal function, and biopsy context. SSc programs may integrate skin biopsy, collagen or TGF-β signatures, matrix neo-epitopes, skin score, high-resolution computed tomography, forced vital capacity, and diffusing capacity according to the intended organ.

### 7.2 Safety-by-design and chronic organ-specific toxicity

Safety-by-design begins with sequence, chemistry, formulation, route, and target biology. Candidate-specific risks include innate sensing, interferon and cytokine induction, complement activation, platelet or hematologic effects, renal or hepatic signals, infusion or injection reactions, local inflammation, anti-carrier immunity, and repeat-dose PK changes (18, 19, 31, 49–52, 110–113). Single-dose tolerability is insufficient for chronic autoimmune use; repeat-dose studies should examine accumulation, altered biodistribution, loss of PD, anti-drug or anti-carrier antibodies, hypersensitivity, organ toxicity, and local tolerability.

Renal and hepatic interpretation requires special care because kidney and liver disease may be part of the target disorder rather than a background comorbidity. Phosphorothioate ASOs commonly distribute to liver and kidney and are degraded slowly by nucleases; renal excretion of intact drug may be limited, so plasma PK alone can underestimate tissue persistence (15, 110). Available case studies suggest that mild-to-moderate renal impairment may have limited effects on plasma exposure for some 2′-O-methoxyethyl (2′-MOE) ASOs, whereas end-stage kidney disease can modestly increase exposure and data in moderate-to-severe hepatic impairment remain sparse (114). These observations cannot be generalized across sequences, conjugates, or carriers.

In LN, baseline proteinuria, hematuria, reduced eGFR, complement consumption, and active urinary sediment can confound attribution of a renal safety signal; trials should prespecify adjudication of disease activity versus drug-related change and include urinary injury markers where appropriate. Hepatocyte-targeted programs require early characterization of hepatic impairment because the liver is both delivery organ and pharmacologic source compartment. SSc adds vascular, blood-pressure, renal-crisis, wound-healing, and pulmonary risks. Regulatory guidance accordingly recommends early assessment of whether kidney or liver function influences disposition, PD, safety, eligibility, or dose, with adequate representation of organ impairment unless scientifically or ethically unjustified (111).

### 7.3 Access, cost, and manufacturing feasibility

Global access cannot be inferred from molecular programmability. Short synthetic oligonucleotides may use modular solid-phase manufacturing, whereas LNP, exosome, membrane-coated, multicomponent, or cell-targeted systems add raw-material, analytical, sterile-processing, release-testing, storage, and batch-reproducibility burdens. Aptamers share major synthesis and impurity-control requirements with other oligonucleotides; extensive stabilization, PEGylation, multimerization, or carrier integration can offset any simplicity relative to antibodies. mRNA programs require template, transcription, purification, encapsulation, particle characterization, potency assays, and cold-chain or stability solutions. Cross-platform acquisition-price comparisons are not currently reliable because marketed indications, dose, frequency, development stage, and health-system contracting differ. Early programs should therefore report dose burden, route, storage, scalable yield, critical quality attributes, and anticipated repeat-dose frequency rather than claiming intrinsic affordability. CMC simplification is a translational objective: every targeting ligand or active co-component should justify its incremental benefit and analytical burden (112, 113).

### 7.4 Trial design and regulatory considerations

First-in-human autoimmune PoM trials should establish compartmental delivery, target engagement, pathway PD, and tolerability before efficacy is interpreted, separating target failure from delivery or endpoint failure.

Eligibility should match the mechanism: complement-active disease for complement programs; interferon-high SLE; B-cell- or plasmablast-active SLE/LN; myeloid-rich synovitis for macrophage-directed RA; stromal/FLS-enriched RA for stromal programs; and active or progressive rather than end-stage fibrosis for SSc.

Dose escalation should be conservative when the target contributes to host defense, or the carrier may activate innate immunity or complement. Sentinel dosing, stopping rules, cytokine and complement monitoring, organ-specific laboratories, and repeat-dose PK/PD should be considered. Background immunosuppression must be controlled because it can obscure pharmacology.

CMC is part of interpretability. Sequence identity, purity and impurities, conjugation consistency, particle attributes, release specifications, stability, sterility, endotoxin, and batch reproducibility can alter exposure, PD, and safety. Current regulatory guidance emphasizes oligonucleotide-specific clinical pharmacology, nonclinical safety, and manufacturing considerations (111–113).

## 8 Discussion

This review advances a falsifiable translational framework rather than another modality catalogue. Building on broad autoimmune-RNA and arthritis-delivery reviews (7–9), it identifies the evidence needed to interpret an early clinical result across target biology, compartmental exposure, intended-cell access, TE, pathway PD, organ response, repeat-dose safety, and manufacturing feasibility. The central cross-disease conclusion is that the same missing observation does not carry the same meaning in every program: its consequence depends on where the RNA is intended to act and which biological link the program claims.

In a hepatocyte-directed program against a liver-derived mediator, direct synovial or renal-cell TE is neither expected nor required; credible interpretation instead requires engagement of the liver-derived target, systemic pathway PD, and a prospectively demonstrated link to synovial or renal biology. Failure to improve the target organ after those upstream steps would weigh against pathway relevance, endotype selection, or the magnitude of systemic modulation. By contrast, in a macrophage-, FLS-, B-cell-, renal-cell-, or lung-fibroblast-directed program, absence of intended-cell TE is a primary interpretive failure: a negative clinical result cannot distinguish an inactive target from inadequate tissue entry, cellular uptake, or intracellular release. Thus, lack of direct TE is a context-dependent limitation rather than a uniform evidentiary deficit.

This distinction explains the disease-specific asymmetry. RA has the largest body of disease-specific nanoparticle and transcript-targeting work, but most remains preclinical and must demonstrate both productive synovial-cell delivery and an advantage over effective therapies. SLE/LN combines strong pathway validation with two additional hazards: therapeutic nucleic acids may amplify innate sensing, and systemic complement suppression can be over-read as renal efficacy unless linked to renal measures. SSc offers the clearest serial tissue-PD window in skin, but skin TE cannot validate lung or other internal-organ pharmacology. Accordingly, biomarker enrichment must be tissue-guided in RA, multi-domain and biopsy-informed where appropriate in SLE/LN, and organ-specific in SSc (53–59, 78, 80–84, 92, 97–101, 103–106, 109). These cross-disease evidence asymmetries and their first go/no-go requirements are summarized in **Table 2**.

**Table 2:** Cross-disease evidence and first go/no-go requirements.

Program	Disease/endotype	Strongest RNA evidence	First go/no-go readout	Interpretation	Key refs
Liver-directed CFB suppression	Complement-active RA or SLE/LN	Human systemic TE/PD outside target disease, including a linked glomerular outcome in IgA nephropathy; RA and LN disease evidence preclinical	CFB/AP suppression linked to synovial or renal response and acceptable infection risk	Class-level PoM tractable; no RA/LN efficacy; AZD6912 discontinued	(73–77, 90)
RA synovial macrophage	Myeloid-rich active synovitis	Multiple disease-relevant animal nanoparticle studies	Intended-cell exposure plus transcript/protein and synovial PD	Conditional on biopsy-enabled, cell-aware delivery	(60–69)
RA FLS/stromal	FLS-rich or stromal-invasive synovitis	Limited disease-specific RNA evidence	FLS—not only macrophage—exposure and invasive/matrix-program modulation	Early and delivery-limited	(54, 56, 70, 71)
SLE interferon/B-cell	Interferon-high or B-cell-active SLE/LN	Human pathway validation; RNA silencing, mRNA and miRNA preclinical	Pathway reduction without RNA-triggered innate activation	Biomarker-enriched PoM; no patient RNA efficacy	(79, 85–89, 91)
LN CFB suppression	Biopsy/biomarker-defined complement-active LN	Factor-B ASO in lupus models; human systemic CFB PD outside LN	Systemic TE/AP PD linked to urinary and renal response	Conditional systemic-target PoM; not direct renal delivery	(76, 77, 90)
SSc skin antifibrotic	Early/progressive cutaneous SSc	Disease-relevant miRNA and mRNA models; human skin PoM outside SSc	Direct RNA/protein engagement plus matrix modulation and local tolerability	Favorable for skin PoM, not internal-organ validation	(97–103, 106–109)
SSc lung fibrosis	Progressive SSc-ILD	Pulmonary-fibrosis and A20 mRNA models	Lung-cell exposure, TE, pulmonary safety and organ-relevant PD	Early and delivery-limited	(104, 105, 107, 109)

AP, alternative pathway; CFB, complement factor B; FLS, fibroblast-like synoviocyte; ILD, interstitial lung disease; LN, lupus nephritis; PD, pharmacodynamic; PoM, proof of mechanism; RA, rheumatoid arthritis; SLE, systemic lupus erythematosus; SSc, systemic sclerosis; TE, target engagement. Interpretations are qualitative and do not predict efficacy, approval, or active candidate status.

Modality and route should therefore be judged by the interpretability they create, not by programmability or uptake alone. miRNA strategies may coordinate macrophage, B-cell, or fibrotic networks, but pleiotropy makes cell context, direct miRNA engagement, gene-set PD, and broad safety indispensable (26–28, 64, 91, 103–108). Local administration may provide a useful PoM window and reduce systemic exposure, yet it does not guarantee intracellular activity and cannot by itself solve disease distribution: a single-joint RA intervention is not systemic disease control, and cutaneous PD is not evidence of pulmonary benefit.

Complement illustrates why pathway and compartment must be interpreted together. In lupus, protective and pathogenic roles vary by pathway, stage, and subgroup; target reduction must therefore be linked to organ-level benefit rather than treated as efficacy itself (3, 4, 78, 90, 92). More broadly, an RNA program is clinically persuasive only if it adds something unavailable from biologics or Janus kinase inhibitors—access to intracellular or non-secreted targets, compartment-restricted exposure, durable but reversible knockdown, coordinated modulation, restoration of a protective protein, reduced treatment burden, or a biomarker-defined combination (80, 81, 85, 86, 93–96, 115). The development decision should therefore turn on whether the proposed modality creates a measurable and clinically relevant causal chain, not simply a novel formulation.

### 8.1 Limitations

Several limitations constrain the certainty rather than the internal logic of this synthesis. The review was narrative, English-language, targeted, and iterative; it used no formal risk-of-bias instrument, quantitative synthesis, or independently duplicated screening, and program status may change after the July 2, 2026, cut-off. Selection and interpretation bias therefore remain possible. The underlying evidence is also uneven: most disease-specific RNA studies are preclinical, animal biodistribution and safety may not translate to human autoimmune tissues, and head-to-head platform, cost, and global-access data are sparse. Finally, the six-domain profiles and PoM descriptors are transparent consensus judgments intended to expose evidentiary gaps; they are not validated scores, comparative efficacy estimates, or probabilities of approval.

## 9 Conclusions

The near-term question is not whether RNA therapeutics are biologically interesting in RA, SLE/LN, or SSc, but whether a program can make its clinical result interpretable. Hepatocyte-directed programs are currently most tractable when engagement of the liver-derived target and systemic PD can be linked prospectively to disease-organ biology; extrahepatic cell-directed programs must first demonstrate productive access and TE in the intended cell. Across diseases, advancement should require a prespecified causal chain from delivery to TE, pathway PD, organ response, and tolerability, together with a clinically meaningful advantage over existing therapy. Programs that cannot establish the missing link should be stopped or redesigned before efficacy is inferred.

## Supplementary material

SUPPLEMENTARY METHODS Documented PubMed/MEDLINE search blocks, the six-domain evidence-appraisal framework with operational definitions and source-hierarchy rules, and the qualitative schematic conventions used in Figures 3–5.

SUPPLEMENTARY TABLE S1 Delivery barriers and mitigation strategies across compartments, with references.

SUPPLEMENTARY TABLE S2 Program-level evidence profile for rheumatoid arthritis.

SUPPLEMENTARY TABLE S3 Program-level evidence profile for systemic lupus erythematosus and lupus nephritis.

SUPPLEMENTARY TABLE S4 Program-level evidence profile and current opportunities for systemic sclerosis.

SUPPLEMENTARY TABLE S5 Full six-domain evidence profile and near-term proof-of-mechanism tractability assessment for all programs reviewed.
