# Supplementary material for: RNA therapeutics in rheumatoid arthritis, systemic lupus erythematosus, and systemic sclerosis: a compartment-aware translational framework
Source: Front Immunol. 2026 Oct 5;17:1950465. doi: 10.3389/fimmu.2026.1950465 (PMC13678641; doi:10.3389/fimmu.2026.1950465)
Supplement: Supplementary file 1 [file Table1.docx]

**Supplementary Material**

**Supplementary Methods. Literature search and evidence-appraisal procedures**

This supplement reports the documented search strategy and the structured appraisal used for this narrative translational review. PubMed/MEDLINE searches were updated through July 2, 2026, without a lower date restriction and were supplemented by ClinicalTrials.gov, regulatory-agency and professional-society documents, major rheumatology conference proceedings, sponsor disclosures, reference-list screening, and targeted searches for recent platform and disease-specific reviews. English-language peer-reviewed primary studies and authoritative guidance were prioritized. Conference abstracts, registries, and sponsor sources were used only for program status or preliminary pharmacology when peer-reviewed reports were unavailable; they were not treated as patient-efficacy evidence. No meta-analysis or formal risk-of-bias instrument was applied.

Evidence was recorded at four non-interchangeable levels: human pathway validation; RNA-platform evidence outside the target disease; disease-specific RNA evidence; and patient efficacy in the target disease. Preclinical studies were appraised for model relevance, route and formulation, intended compartment and cell, direct target engagement, pathway pharmacodynamics (PD), component controls, repeat dosing, toxicity, and human translatability. Source hierarchy was: peer-reviewed human evidence in the target disease; peer-reviewed human platform evidence outside the target disease; disease-relevant animal studies; mechanistic or in vitro studies; and registry/regulatory/sponsor material for status only.

Two or more authors discussed every program profile and agreed the final wording by consensus. The exercise was not a formal independent duplicate rating. Discordant studies were not averaged: differences in sequence, carrier, route, model, compartment, and endpoint were retained; the evidence category was limited by the most relevant credible counterevidence or missing translational link. A single positive model did not override absent intended-cell delivery, absent target engagement, contradictory safety findings, or failure in a more clinically relevant setting.

**Documented PubMed/MEDLINE search blocks**

| Search block | Exact query |
| --- | --- |
| Rheumatoid arthritis | ("Arthritis, Rheumatoid"[Mesh] OR "rheumatoid arthritis"[tiab]) AND ("antisense oligonucleotide*"[tiab] OR antisense[tiab] OR siRNA[tiab] OR "small interfering RNA"[tiab] OR "RNA interference"[tiab] OR microRNA*[tiab] OR miRNA*[tiab] OR antagomiR*[tiab] OR "microRNA mimic*"[tiab] OR mRNA[tiab] OR "messenger RNA"[tiab] OR "RNA aptamer*"[tiab] OR aptamer*[tiab]) AND (therap*[tiab] OR deliver*[tiab] OR nanoparticle*[tiab] OR exosome*[tiab] OR GalNAc[tiab] OR "lipid nanoparticle*"[tiab] OR biodistribution[tiab] OR "endosomal escape"[tiab] OR "target engagement"[tiab] OR pharmacodynamic*[tiab] OR safety[tiab] OR trial[tiab]) |
| Systemic lupus erythematosus / lupus nephritis | ("Lupus Erythematosus, Systemic"[Mesh] OR "Lupus Nephritis"[Mesh] OR "systemic lupus erythematosus"[tiab] OR "lupus nephritis"[tiab]) AND ("antisense oligonucleotide*"[tiab] OR antisense[tiab] OR siRNA[tiab] OR "small interfering RNA"[tiab] OR "RNA interference"[tiab] OR microRNA*[tiab] OR miRNA*[tiab] OR antagomiR*[tiab] OR "microRNA mimic*"[tiab] OR mRNA[tiab] OR "messenger RNA"[tiab] OR "RNA aptamer*"[tiab] OR aptamer*[tiab]) AND (therap*[tiab] OR deliver*[tiab] OR nanoparticle*[tiab] OR exosome*[tiab] OR GalNAc[tiab] OR "lipid nanoparticle*"[tiab] OR biodistribution[tiab] OR "endosomal escape"[tiab] OR "target engagement"[tiab] OR pharmacodynamic*[tiab] OR safety[tiab] OR trial[tiab]) |
| Systemic sclerosis | ("Scleroderma, Systemic"[Mesh] OR "systemic sclerosis"[tiab] OR scleroderma[tiab]) AND ("antisense oligonucleotide*"[tiab] OR antisense[tiab] OR siRNA[tiab] OR "small interfering RNA"[tiab] OR "RNA interference"[tiab] OR microRNA*[tiab] OR miRNA*[tiab] OR antagomiR*[tiab] OR "microRNA mimic*"[tiab] OR mRNA[tiab] OR "messenger RNA"[tiab] OR "RNA aptamer*"[tiab] OR aptamer*[tiab]) AND (therap*[tiab] OR deliver*[tiab] OR nanoparticle*[tiab] OR exosome*[tiab] OR GalNAc[tiab] OR "lipid nanoparticle*"[tiab] OR biodistribution[tiab] OR "endosomal escape"[tiab] OR "target engagement"[tiab] OR pharmacodynamic*[tiab] OR safety[tiab] OR trial[tiab]) |
| Cross-cutting delivery, PD, and safety | (oligonucleotide*[tiab] OR siRNA[tiab] OR mRNA[tiab] OR microRNA*[tiab] OR aptamer*[tiab]) AND (GalNAc[tiab] OR "lipid nanoparticle*"[tiab] OR LNP[tiab] OR delivery[tiab] OR biodistribution[tiab] OR "endosomal escape"[tiab] OR immunogenicity[tiab] OR "complement activation"[tiab] OR "target engagement"[tiab] OR pharmacodynamic*[tiab]) |

**Operational six-domain evidence appraisal**

| Domain | Categories | Operational requirement | Discordance / escalation rule | Do not equate |
| --- | --- | --- | --- | --- |
| Human disease-biology validation | Limited / indirect / strong | Limited: single, small, or indirect human signal. Indirect: replicated human association without target-disease intervention. Strong: convergent human genetics/tissue/biomarker evidence and/or intervention against the pathway in the target disease. | RNA delivery or efficacy is never inferred from pathway validation. | Biologic efficacy ≠ RNA-drug validation. |
| Disease-specific RNA evidence | Conceptual / in vitro / disease-relevant animal / human | Highest category requires an RNA intervention in the named disease setting. Animal status requires a disease-relevant model with RNA-component attribution. | Combination-platform benefit is not assigned to the RNA component without component controls. | Fibrosis evidence outside SSc ≠ SSc efficacy. |
| Relevant-compartment delivery | Unproven / preclinical / human indirect / human direct | Direct requires measured exposure in the intended human tissue and, when central to the claim, cell population plus productive access. Indirect permits a justified systemic surrogate only. | General platform experience is labeled outside-disease and cannot upgrade an extrahepatic program. | Hepatocyte GalNAc maturity ≠ synovial, renal-cell, skin, or lung delivery. |
| Human RNA target engagement / PD | None / indirect / direct | Direct requires a prespecified target-specific molecular change after the RNA intervention. Indirect is downstream pathway modulation without direct molecular engagement. | In-disease and outside-disease human PD are reported separately. | Circulating CFB PD ≠ synovial or renal efficacy. |
| Patient efficacy | None / exploratory / controlled | Exploratory denotes uncontrolled or early patient signal. Controlled requires prospective comparative evidence for a clinical or organ endpoint in the target disease. | No category is inferred from animal benefit, target engagement, or non-RNA therapy. | PD ≠ organ response ≠ patient benefit. |
| Repeat-dose safety / CMC | Early / intermediate / mature platform with candidate gaps | Early: limited repeat-dose and scale-up data. Intermediate: repeat-dose nonclinical or early human package with defined critical quality attributes. Mature platform: substantial clinical/marketed class experience, while sequence-, carrier-, target-, route-, organ-, and indication-specific gaps remain. | The lower of repeat-dose safety or manufacturability constrains the program-level description. | Platform maturity ≠ candidate or disease-program maturity. |

*Near-term proof-of-mechanism (PoM) synthesis: ‘favorable’ means that route, target engagement and a disease-relevant PD window can support an interpretable early human study; ‘conditional’ names a required missing capability; ‘early/delivery-limited’ indicates that human compartment delivery or direct target engagement is absent. These terms do not predict efficacy, approval, active-candidate status, or commercial value, and no numerical weighting was used.*

**Qualitative schematic conventions used in Figures 3 to 5**

The segmented bars in Figures 3 to 5 are consensus-based qualitative schematic markers used only to indicate the broad delivery/access position of the named compartment or cell population. They are visually distinct from the circle and triangle markers used within the cards to denote evidence type. The bars are not measurements, scores, effect estimates, validated maturity categories, or probabilities of success; the visual distance between one and four filled segments has no quantitative meaning. Four filled segments: delivery to the named population is achieved with a platform already in chronic human use in some indication, and the limiting step lies downstream of uptake. Three filled segments: the population is reachable but not selectively, or cell-restricted delivery has been attempted rather than assumed. Two filled segments: delivery has been attempted only with a specialized or organ-directed platform, with model-level evidence. One filled segment: no direct delivery to the named compartment or cell population has been demonstrated; displayed entries may therefore be conceptual, or supported only by disease-model evidence without compartment-specific delivery. These descriptors support narrative ordering only. They do not state efficacy or pharmacodynamic accessibility, and a card may show four filled segments while having no human evidence in the disease. The categories are not derived from measurement and should not be compared across figures beyond the qualitative definitions given here.

**Supplementary Table S1. Cross-cutting delivery, chronic-safety, access, and CMC barriers**

| Barrier | Most relevant context | Why it matters | Required evidence/readout | Mitigation / decision rule | Key refs |
| --- | --- | --- | --- | --- | --- |
| Exposure / half-life | ASO, siRNA, mRNA, aptamers | Nuclease degradation, renal clearance, protein binding, or reticuloendothelial uptake can shorten or redistribute exposure. | Plasma/tissue PK, intact drug, protein binding, stability, dose burden | Chemistry, conjugation, protective carrier; aptamer capping, sugar modification, multimerization or half-life extension | (11,13–17,26,29,30,33) |
| Hepatocyte boundary | Liver-derived complement | GalNAc can support hepatocyte delivery and systemic target suppression but not direct synovial or renal-cell exposure. | Circulating target/AP PD linked prospectively to organ response | Use only when hepatic production is causal; label organ effect as indirect | (24,37,38,73–77,90) |
| Extrahepatic cell delivery | RA macrophage/FLS; SLE B cell; SSc fibroblast | Organ uptake can reflect phagocytic sequestration or non-productive uptake in the wrong cell. | Sorted-cell/single-cell exposure and direct target engagement | Receptor mapping, ligand validation, local route, cell-aware biodistribution | (39–48,50–52,60–71,79,85–91) |
| Endosomal escape | siRNA and mRNA | Internalization without cytosolic release does not support RISC activity or translation. | Cytosolic activity assay plus target-specific RNA/protein change | Ionizable, pH-responsive or membrane-active components with toxicity controls | (41–46) |
| Renal disease / impairment | LN and chronic oligonucleotide dosing | Proteinuria, eGFR change and urinary abnormalities can reflect active LN, tissue exposure, or toxicity; plasma PK may not capture renal persistence. | Prespecified renal adjudication, urinary injury markers, eGFR/proteinuria trajectory, organ-impairment PK/PD | Represent renal impairment when justified; distinguish disease activity from drug injury | (15,78,81,111,114) |
| Hepatic disease / impairment | Hepatocyte-directed and systemically distributed programs | The liver may be both delivery organ and pharmacologic source; moderate/severe impairment data are sparse and non-generalizable. | Liver tests, hepatic-impairment exposure/PD, tissue-persistence and dose rationale | Candidate-specific evaluation; avoid class-wide PK extrapolation | (15,111,114) |
| Innate sensing / complement | Especially SLE/LN; systemic carriers | TLR/cytosolic sensing or carrier-associated complement activation may mimic or intensify autoimmune inflammation. | Human-cell interferon/cytokines; C3a, C5a, sC5b-9; clinical reactions | Motif/chemistry and formulation screening; sentinel dosing and stopping rules | (12,13,18,19,31,48–52) |
| Repeat-dose carrier immunity | PEG-, lipid-, polymer- or biomimetic systems | Antibodies or immune memory may alter PK, reduce PD, or cause hypersensitivity. | Anti-carrier assays, repeat-dose PK/PD, cytokines, complement, local tolerability | Simplify carrier; justify repeat interval and stealth component | (49–52) |
| Fibrotic penetration / organ specificity | SSc skin and lung | Dense matrix and vascular injury can restrict access; skin PD is not a surrogate for lung or vascular benefit. | Cell-aware biopsy PD; HRCT/PFT and organ-specific safety for lung | Skin-local PoM where appropriate; validate pulmonary delivery separately | (97–109) |
| CMC, access, and cost | All; highest burden for multicomponent carriers | Added ligands, active co-components, exosomes or membrane coatings increase analytical, sterile-processing, storage and batch-reproducibility demands. Cross-platform acquisition-price comparisons are not reliable. | Yield, dose/frequency, route, storage, critical quality attributes, release tests, stability, reproducibility | Minimum-complexity design; justify each component and scalable process | (112,113) |

*AP, alternative pathway; ASO, antisense oligonucleotide; CMC, chemistry, manufacturing, and controls; eGFR, estimated glomerular filtration rate; FLS, fibroblast-like synoviocyte; GalNAc, N-acetylgalactosamine; HRCT, high-resolution computed tomography; LN, lupus nephritis; LNP, lipid nanoparticle; mRNA, messenger RNA; PD, pharmacodynamic; PEG, polyethylene glycol; PFT, pulmonary function testing; PK, pharmacokinetic; PoM, proof of mechanism; RA, rheumatoid arthritis; RISC, RNA-induced silencing complex; siRNA, small interfering RNA; SLE, systemic lupus erythematosus; SSc, systemic sclerosis; TLR, Toll-like receptor.*

**Supplementary Table S2. Directly traceable RNA programs in rheumatoid arthritis**

| Program / target | Modality, carrier, route, intended compartment | Study type / model | Reported or required TE/PD | Principal limitation / synthesis | Key refs |
| --- | --- | --- | --- | --- | --- |
| TNF silencing | siRNA; chitosan/polymer or LNP; systemic/local; inflamed synovium/macrophage | Experimental arthritis | Reduced arthritis and TNF-associated inflammatory readouts; human synovial TE absent | Must outperform effective anti-TNF therapy; conditional local PoM | (60,61) |
| IL-1β silencing | siRNA; macrophage-biased lipidoid–polymer NP; synovial myeloid cells | Mouse arthritis | Reduced disease with macrophage/cytokine PD | IL-1β is not a dominant broad-RA clinical axis; endotype-specific | (62) |
| RELA/p65 NF-κB | siRNA; peptide/hybrid nanocarriers; local/cell-directed | Experimental arthritis | p65/pathway suppression and reduced inflammation | Broad NF-κB inhibition and host defense; conditional on local/cell specificity | (67,68) |
| BTK | siRNA NP; inflammatory immune compartment | Preclinical RA model | BTK knockdown with B-cell/myeloid inflammatory improvement | Cell specificity, redundancy, infection; early | (69) |
| NOTCH1 | siRNA NP ± methotrexate; synovial stromal/inflammatory cells | Collagen-induced arthritis | NOTCH1/pathway suppression with disease improvement | FLS-specific delivery and pleiotropy remain unresolved | (70,71) |
| GSDME + 4-octyl itaconate | siRNA plus active small molecule; folate/macrophage-membrane hybrid NP | Dual-component arthritis model | GSDME knockdown, metabolic PD and joint improvement reported | RNA attribution, interaction and CMC complexity; early | (63) |
| Complement factor B | GalNAc-siRNA or ASO; hepatocyte; systemic complement | RA/NHP platform data; healthy-participant ASO phase I and IgA nephropathy phase II outside RA | Human CFB and AP suppression outside RA, with a linked glomerular outcome in IgA nephropathy; no synovial TE or RA efficacy | AZD6912 discontinued; complement-active selection and infection safety required | (73–77) |
| miR-155 | miRNA inhibitor; preferably macrophage-directed/local | Human RA biology + experimental arthritis | miRNA and macrophage gene-set engagement required; disease benefit preclinical | Pleiotropy across myeloid/lymphoid cells; conditional | (64) |
| miR-124 + methotrexate | miRNA mimic in methotrexate-conjugated hybrid micelle | Combination arthritis model | Gene-network PD and disease improvement reported | Two-component attribution and reproducibility; early | (65) |
| PTEN mRNA + TNF decoy | mRNA in engineered macrophage-membrane NP with active decoy | Dual-mechanism arthritis model | Encoded PTEN, localization and joint PD reported | Cannot assign benefit to PTEN alone; carrier immunity/CMC | (66) |
| Synovial endotype strategy | Biopsy/fluid/imaging/cell-aware transcriptomics; no single RNA product | Human tissue and biopsy-driven trials | Defines pathway-active cells and a tissue PD window | Sampling burden and assay standardization; favorable enabling principle | (53–59) |

*AP, alternative pathway; ASO, antisense oligonucleotide; BTK, Bruton tyrosine kinase; CFB, complement factor B; CMC, chemistry, manufacturing, and controls; FLS, fibroblast-like synoviocyte; GalNAc, N-acetylgalactosamine; GSDME, gasdermin E; IgA, immunoglobulin A; IL-1β, interleukin-1 beta; LNP, lipid nanoparticle; mRNA, messenger RNA; miRNA, microRNA; NF-κB, nuclear factor kappa B; NHP, non-human primate; NP, nanoparticle; PD, pharmacodynamic; PoM, proof of mechanism; PTEN, phosphatase and tensin homolog; RA, rheumatoid arthritis; siRNA, small interfering RNA; TE, target engagement; TNF, tumor necrosis factor.*

**Supplementary Table S3. Directly traceable RNA programs in SLE and lupus nephritis**

| Program / target | Modality, carrier, route, intended compartment | Study type / model | Reported or required TE/PD | Principal limitation / synthesis | Key refs |
| --- | --- | --- | --- | --- | --- |
| IRF5 | Gene-silencing RNA; systemic/renal immune compartments | Preclinical LN | IRF5 knockdown and interferon-signature reduction with renal PD | Delivery, durability, host defense and RNA-triggered innate activation; early | (87) |
| IRF5 + BLyS/BAFF | Dual-pathway RNA silencing | Preclinical LN combination | Attributable knockdown of both axes and renal/immune PD required | Dose attribution and broad immune effects; early/complex | (87) |
| Anti-BAFF mRNA | Anti-CD19-engineered exosomes; B-cell delivery and local antibody expression | Lupus mice | B-cell delivery, encoded antibody kinetics, BAFF neutralization and renal PD | Exosome CMC, off-target transfection, expression control and immunogenicity | (88) |
| CD40 | Cholesterol-conjugated siRNA; systemic/renal exposure | Preclinical LN | CD40 RNA/protein reduction linked to serologic and renal PD | Protective immunity, infection and cell specificity; early | (89) |
| Complement factor B | Liver-directed ASO; systemic AP suppression, not direct renal-cell delivery | Lupus models; healthy-participant and IgA nephropathy human CFB PD outside LN | Human systemic CFB/AP suppression outside LN, with proteinuria reduction in IgA nephropathy; renal response in LN only preclinical | Complement-stage heterogeneity, infection and disease/organ attribution; conditional | (76,77,90) |
| miR-7 | B-cell-targeted polymer NP; miRNA inhibitor | Preclinical lupus | Direct miR-7 and B-cell gene-set PD with autoantibody/renal response | Pleiotropy and durable B-cell specificity; early | (91) |
| Immune-silent design | Sequence/chemistry/formulation screening; all routes | Platform-level human and nonclinical evidence | Prespecified interferon, cytokine and complement limits | Mandatory gate in nucleic-acid-sensing disease; not a separate efficacy program | (12,13,18,19,31,48–52) |

*AP, alternative pathway; ASO, antisense oligonucleotide; BAFF, B-cell activating factor; BLyS, B-lymphocyte stimulator; CD19, cluster of differentiation 19; CD40, cluster of differentiation 40; CFB, complement factor B; CMC, chemistry, manufacturing, and controls; IgA, immunoglobulin A; IRF5, interferon regulatory factor 5; LN, lupus nephritis; mRNA, messenger RNA; miRNA, microRNA; NP, nanoparticle; PD, pharmacodynamic; siRNA, small interfering RNA; SLE, systemic lupus erythematosus; TE, target engagement.*

**Supplementary Table S4. Directly traceable RNA programs in systemic sclerosis**

| Program / target | Modality, carrier, route, intended compartment | Study type / model | Reported or required TE/PD | Principal limitation / synthesis | Key refs |
| --- | --- | --- | --- | --- | --- |
| miR-29 replacement—skin | miRNA mimic; skin-local/intradermal or validated systemic carrier | SSc biology; human skin PoM outside SSc | Direct miR-29 engagement and matrix-transcript/biopsy PD | SSc efficacy, durability and wound-healing safety unproven; conditional skin PoM | (102,103) |
| miR-29 replacement—lung | miRNA mimic; inhaled/specialized lung carrier | Pulmonary-fibrosis models, not SSc-ILD efficacy | Lung-cell exposure, miR-29/matrix PD, HRCT/PFT | Pulmonary delivery and repeat-dose safety; early/delivery-limited | (104,105) |
| miR-21 inhibition | Anti-miRNA expression plasmid/miRZip by hydrodynamic delivery; not formulated antagomiR | Bleomycin mouse model | miR-21 and STAT3/PTEN-related PD with fibrosis improvement | Non-translatable route, plasmid expression and pleiotropy; biological PoC only | (107) |
| miR-155 inhibition | Topical/skin-local antagomiR | Experimental scleroderma | miR-155 engagement, collagen, α-SMA and pathway PD | Broad immune role; systemic inhibition risk; early/local | (108) |
| TNFAIP3/A20 restoration | mRNA-LNP; dermal fibroblasts and weekly intramuscular mouse dosing | SSc-relevant preclinical study | Encoded A20, TRAF6/NF-κB and DREAM–SMAD2 PD; skin/lung fibrosis | No human delivery/PD; cell specificity, duration and non-target expression | (109) |
| TGF-β/SMAD, CCN2/CTGF, collagen | ASO/siRNA concepts; fibroblast/skin/local delivery | Human pathway/transcriptomics; limited disease-specific RNA | Direct transcript knockdown plus matrix PD required | Redundancy, wound healing and fibroblast-specific delivery; early | (97–101) |
| IL-6/JAK/STAT or immune transcripts | ASO/siRNA concepts; immune/tissue-directed | Non-RNA pathway validation; sparse SSc RNA evidence | Inflammatory signature plus fibroblast/organ PD required | Host defense and delivery specificity; conceptual/early | (96,98–100) |
| Vascular-protective programs | mRNA/miRNA/ASO concepts; vascular delivery | Mostly conceptual in SSc RNA therapeutics | Endothelial PD, digital-ulcer/vascular measures and renal/pulmonary safety | Immature delivery and non-target-expression risk; conceptual | (5,6,93,99) |
| Skin PD strategy | Serial skin biopsy, blood matrix markers and imaging; no single RNA product | Human translational studies | Skin transcriptomics, neo-epitopes, histology and mRSS | Internal-organ surrogacy is unproven; favorable enabling strategy only | (97–103,106) |

*A20, tumor necrosis factor alpha-induced protein 3; ASO, antisense oligonucleotide; CCN2, cellular communication network factor 2; CTGF, connective tissue growth factor; DREAM, downstream regulatory element antagonist modulator; HRCT, high-resolution computed tomography; IL-6, interleukin 6; JAK, Janus kinase; LNP, lipid nanoparticle; mRNA, messenger RNA; miRNA, microRNA; mRSS, modified Rodnan skin score; NF-κB, nuclear factor kappa B; PD, pharmacodynamic; PFT, pulmonary function testing; PoC, proof of concept; PoM, proof of mechanism; PTEN, phosphatase and tensin homolog; siRNA, small interfering RNA; SMAD2, SMAD family member 2; SSc, systemic sclerosis; SSc-ILD, systemic sclerosis-associated interstitial lung disease; STAT, signal transducer and activator of transcription; STAT3, signal transducer and activator of transcription 3; TE, target engagement; TGF-β, transforming growth factor beta; TNFAIP3, tumor necrosis factor alpha-induced protein 3; TRAF6, TNF receptor-associated factor 6; α-SMA, alpha-smooth muscle actin.*

**Supplementary Table S5. Transparent application of the six-domain framework**

| Program and disease/endotype | Human biology | Disease-specific RNA evidence | Relevant delivery | Human RNA TE/PD | Patient efficacy | Repeat-dose safety/CMC and cautious synthesis (key refs) |
| --- | --- | --- | --- | --- | --- | --- |
| ****Liver CFB suppression — complement-active RA/LN**** | Strong but stage- and endotype-dependent | Surrogate CFB siRNA reduced arthritis in murine CAIA; factor-B ASO improved murine LN | Mature hepatocyte-directed route; NHP and human platform data support systemic CFB suppression, not direct synovial or renal-cell delivery | Human systemic CFB/AP suppression demonstrated outside RA/LN | No efficacy evidence in RA or LN; an exploratory proteinuria signal was reported in IgA nephropathy outside the target diseases | Candidate-specific infection and over-suppression gaps; favorable only for systemic-PD-focused PoM (73–77,90) |
| ****RA synovial macrophage — myeloid-rich synovitis**** | Strong human tissue rationale | Multiple disease-relevant animal siRNA, miRNA, mRNA and combination-platform studies | Preclinical macrophage uptake and target modulation are achievable, but cell-resolved productive activity in human RA synovium remains unproven | None in patients with RA | None | Immature formulation and repeat-dose package; conditional on biopsy-enabled, cell-aware PoM (60–69) |
| ****RA FLS/stromal — FLS-rich synovitis**** | Strong human tissue rationale | Limited disease-specific RNA evidence; principally Notch-related models | Less mature than macrophage delivery; FLS exposure remains unproven | None in patients with RA | None | Early CMC/safety; delivery-limited (54–56,70,71) |
| ****SLE interferon — interferon-high SLE/LN**** | Strong genetic, human and non-RNA pathway validation | IRF5 silencing improved disease-relevant outcomes in lupus models | Systemic immune-cell delivery remains unvalidated in humans | None in patients with SLE/LN | None | High innate-sensing and host-defense concern; conditional biomarker-enriched PoM (79,85–87) |
| ****SLE/LN B-cell/co-stimulation — B-cell-active disease**** | Strong BAFF and co-stimulatory biology | Dual IRF5/BAFF silencing, anti-BAFF-antibody mRNA delivery, CD40 siRNA and anti-miR-7 in lupus models | Cell-targeted delivery remains preclinical | None in patients with SLE/LN | None | Exosome/particle CMC, expression-control and infection gaps; early targeted PoM (87–89,91) |
| ****LN complement — complement-active LN**** | Strong but biologically dual and stage-dependent | Factor-B ASO improved renal and survival outcomes in murine LN | Hepatocyte-directed route; not direct renal-cell delivery | Human systemic CFB/AP suppression demonstrated outside LN | No efficacy evidence in LN; an exploratory proteinuria signal was reported in IgA nephropathy | Infection and immune-complex-handling risks; conditional systemic-target PoM (76,77,90) |
| ****SSc skin antifibrotic — early/progressive cutaneous SSc**** | Strong fibrosis biology with molecularly defined subsets | miR-21/miR-155 and A20 models; miR-29 human skin PoM outside SSc | Skin is accessible, but RNA delivery to activated fibroblasts in human SSc skin remains unproven | Human skin pathway PD demonstrated outside SSc; none in SSc | None in SSc | Route-specific wound-healing and chronic-safety gaps; favorable for skin PoM only (97–103,107–109) |
| ****SSc lung fibrosis — progressive SSc-ILD**** | Strong unmet need and fibrosis biology | Pulmonary miR-29 and A20 models | Specialized pulmonary delivery remains preclinical and unvalidated in SSc | None in patients with SSc-ILD | None | Pulmonary inflammation, infection and repeat-dose gaps; early/delivery-limited (104,105,109) |
| ****miRNA network modulation — selected RA/SLE/SSc cell states**** | Strong but cell- and context-dependent | Multiple disease-relevant preclinical studies | Carrier-, route- and cell-target-dependent | Limited human PD outside the target diseases; no in-disease human TE/PD | None in the target diseases | Pleiotropy; conditional on direct miRNA engagement and an accessible PD window (64,65,91,102–108) |
| ****mRNA restoration — RA local, SLE B cell and SSc A20 programs**** | Mechanistically plausible | PTEN mRNA/TNF-decoy platform in experimental arthritis; anti-BAFF-antibody mRNA exosomes in lupus models; A20 mRNA-LNP in SSc models | Local or cell-targeted delivery remains preclinical | None in the target diseases | None | Expression control, carrier immunity, repeat-dose and CMC gaps; early (66,88,109) |

*A20, tumor necrosis factor alpha-induced protein 3; AP, alternative pathway; ASO, antisense oligonucleotide; BAFF, B-cell activating factor; CAIA, collagen antibody-induced arthritis; CD40, cluster of differentiation 40; CFB, complement factor B; CMC, chemistry, manufacturing, and controls; FLS, fibroblast-like synoviocyte; IgA, immunoglobulin A; IRF5, interferon regulatory factor 5; LN, lupus nephritis; LNP, lipid nanoparticle; mRNA, messenger RNA; miRNA, microRNA; NHP, non-human primate; PD, pharmacodynamic; PoM, proof of mechanism; PTEN, phosphatase and tensin homolog; RA, rheumatoid arthritis; siRNA, small interfering RNA; SLE, systemic lupus erythematosus; SSc, systemic sclerosis; SSc-ILD, systemic sclerosis-associated interstitial lung disease; TE, target engagement; TNF, tumor necrosis factor. Categories are structured qualitative judgments assigned by author consensus, not validated scores, formal risk-of-bias ratings, efficacy rankings, active-candidate claims or approval predictions. Human RNA TE/PD outside the named disease is reported separately from patient efficacy in the target disease.*
